# Peripheral vascular function, including endothelium‐dependent measures, and dementia risk: The Framingham Heart Study

**DOI:** 10.1002/alz.71396

**Published:** 2026-04-28

**Authors:** Qiushan Tao, Jingyan Han, Ting Fang Alvin Ang, Lei Hou, Chunyu Liu, Joanne M. Murabito, Kathryn L. Lunetta, Jesse Mez, Michael L. Alosco, Thor D. Stein, Xiaoling Zhang, Rhoda Au, Lindsay Farrer, Joseph N. Palmisano, Naomi M. Hamburg, Wei Qiao Qiu

**Affiliations:** ^1^ Department of Pharmacology, Physiology & Biophysics Boston University Chobanian & Avedisian School of Medicine Boston Massachusetts USA; ^2^ Department of Medicine Boston University Chobanian & Avedisian School of Medicine Boston Massachusetts USA; ^3^ Department of Anatomy & Neurobiology Boston University Chobanian & Avedisian School of Medicine Boston Massachusetts USA; ^4^ Departments of Biostatistics Boston University Chobanian & Avedisian School of Public Health Boston Massachusetts USA; ^5^ Departments of Epidemiology Boston University Chobanian & Avedisian School of Public Health Boston Massachusetts USA; ^6^ Framingham Heart Study Boston University Chobanian & Avedisian School of Medicine Boston Massachusetts USA; ^7^ Department of Neurology Boston University Chobanian & Avedisian School of Medicine Boston Massachusetts USA; ^8^ Boston University Alzheimer's Disease Research Center and BU CTE Cetner Boston University Chobanian & Avedisian School of Medicine Boston Massachusetts USA; ^9^ VA Boston Healthcare System Boston Massachusetts USA; ^10^ Department of Psychiatry Boston University Chobanian & Avedisian School of Medicine Boston Massachusetts USA

**Keywords:** Alzheimer's disease (AD), brachial artery flow‐mediated dilation (FMD), dementia, endothelial function, microvascular function, mild cognitive impairment (MCI), reactive hyperemia (RH)

## Abstract

**INTRODUCTION:**

The relationship between peripheral vascular health, including endothelia, cognitive decline, and Alzheimer's disease (AD) dementia risk is unclear.

**METHODS:**

In this study, 2844 dementia‐free Framingham Offspring participants (mean age 60.6 years, 53.2% women) had baseline brachial artery flow‐mediated dilation (FMD%) and reactive hyperemia (RH). Participants were then followed for a median of 17 years for incident AD and underwent plasma biomarker testing and brain magnetic resonance imaging.

**RESULTS:**

FMD% (hazard ratio [HR] = 0.83, 95% confidence interval [CI] 0.76 to 0.91, *p* < 0.001) and RH (HR = 0.89, 95% CI 0.79 to 0.99, *p* = 0.049) were negatively associated with incident AD dementia after adjusting for confounders. Associations were stronger in individuals with elevated C‐reactive protein. Poor vascular function correlated with higher plasma AD biomarkers, smaller brain volumes, greater white matter injury, and increased cerebral microbleeds.

**DISCUSSION:**

Poor FMD% and RH may serve as a prognostic biomarker for cerebrovascular pathology, including endothelial dysfunction in the AD brain.

## Highlights

- Lower FMD and RH are linked to higher risk of AD dementia, and the associations are stronger in individuals with systemic inflammation.
- Poor vascular function relates to higher plasma AD biomarkers.
- Vascular dysfunction correlates with brain atrophy and microbleeds.

## 1 BACKGROUND

Cerebrovascular dysfunction is increasingly recognized as an early and contributing factor in the pathogenesis of Alzheimer's disease (AD).^1^, ^2^, ^3^, ^4^ Abnormalities in the brain's vasculature including the endothelium, the monolayer of cells lining blood vessels, can impair nutrient and oxygen delivery, disrupt blood–brain barrier (BBB) integrity, and promote neuroinflammation, thereby accelerating neurodegeneration. The endothelium plays a central role in maintaining vascular homeostasis and cerebral perfusion, and emerging evidence suggests that endothelial dysfunction is not merely a consequence but also an early driver of AD pathology.^5^, ^6^

Flow‐mediated dilation (FMD), a non‐invasive ultrasound‐based measure of brachial artery dilation in response to increased blood flow, is a well‐established method for assessing systemic vascular including endothelial function and predicting cardiovascular disease (CVD) risk.^7^ The accompanying reactive hyperemia (RH) response, which is characterized by the transient surge in blood flow and velocity following cuff‐induced ischemia, reflects microvascular reactivity in resistance vessels. Given the known contribution of cerebral small vessel disease to AD, ^8^ these peripheral vascular measures may provide insight into early vascular dysfunction in the brain for AD risk. It is possible that peripheral vascular dysfunction may mirror or contribute to cerebrovascular injury in AD. Although prior population‐based studies have examined peripheral vascular function in relation to cognitive outcomes, findings have been inconsistent. For example, one cross‐sectional study with a small sample size found that low FMD was associated with mild cognitive impairment (MCI)^9^, ^10^; on the other hand, the Cardiovascular Health Study^11^ evaluated brachial artery FMD in an older and racially diverse cohort and did not observe a statistically significant association with AD dementia. Importantly, few studies have investigated whether peripheral vascular measures, such as FMD and RH, relate to preclinical AD stages, for example, cognitive decline, or to plasma and imaging biomarkers that reflect underlying AD pathology. Thus, whether impaired peripheral vascular function serves as an early, measurable indicator of AD‐related cerebrovascular injury remains unclear.

Phosphorylated tau (p‐tau) species have emerged as a driver of vascular dysfunction in AD. Soluble tau has been shown to enter brain endothelial cells and impair their function,^12^ and our earlier studies demonstrated the accumulation of p‐tau (including p‐tau181, p‐tau217, and p‐tau231) in the cerebral vasculature of AD brains.^13^, ^14^ In parallel, circulating total tau (t‐tau) and the plasma Aβ42/40 ratio are widely used biomarkers that, while not disease‐specific, reflect tau‐related neurodegeneration and amyloid pathology, respectively, and provide complementary information on AD‐related pathophysiological processes.^15^, ^16^ These findings support a bidirectional relationship between tau pathology and vascular injury, reinforcing the vascular hypothesis of AD. Yet, despite the established link between endothelial health and cognitive decline, no validated clinical tools currently exist to assess brain endothelial function in vivo for AD. Multiple studies have demonstrated associations between vascular physiological measures and magnetic resonance imaging (MRI)‐derived neuroimaging markers, including regional brain volumes, particularly hippocampal atrophy, and markers of vascular brain injury, such as white matter hyperintensities (WMHs) and cerebral microbleeds (CMBs), which are commonly implicated in AD.^17^, ^18^ Accordingly, examining the relationships between FMD, RH, and these neuroimaging biomarkers may provide insight into vascular contributions to AD risk.

The Framingham Heart Study (FHS) had the data of FMD percentage (FMD%) and RH measurements in Generation 2 (Gen 2) participants between 1998 and 2001, alongside detailed vascular, cognitive, and biomarker assessments.^19^ We used the FHS cohort and examined whether FMD% and RH were associated with incident AD dementia and MCI and whether these vascular measures correlated with plasma biomarkers of AD, including plasma biomarkers, p‐tau, and amyloid beta (Aβ), and neuroimaging biomarkers. Identifying a non‐invasive, widely available vascular biomarker of AD risk could enable earlier detection and prevention efforts, bridging peripheral vascular function and neurodegenerative disease frameworks in mechanism investigation and clinical care.

## 2 METHODS

### 2.1 Study design and participants

The FHS cohort was initiated by the National Heart, Lung, Blood Institute (NHLBI) to identify determinants of CVD and stroke. The FHS first recruited Generation 1 (Gen 1; *n* = 5209) in 1948. Since then, FHS has expanded to include the offspring of Gen 1 (Gen 2; *n* = 5124, since 1971).^20^ This study utilized data from the FHS Offspring Cohort (Generation 2, Exam 7 1998 to 2001; longitudinal follow‐up 1998 to 2024). A total of 2844 Gen 2 participants with baseline measures of FMD and RH measured at exam 7 (1998 and 2001) were included. Participants were followed for up to an average of 17.5 ± 6.2 years to assess incident MCI, AD dementia, and all‐cause dementia (Figure S1). Additional assessments included repeated neuropsychological (NP) test scores, plasma AD biomarkers, and neuroimaging measurements.

The FHS was approved by the Institutional Review Board (IRB) of Boston University School of Medicine and conducted in accordance with the ethical standards of the 1964 Declaration of Helsinki and its later amendments or comparable ethical standards. All participants provided written informed consent prior to participation.

**RESEARCH IN CONTEXT**

1. **Systematic review**: We reviewed the existing literature on the relationship between vascular health and AD, with particular attention to the potential contributions of cerebrovascular and brain endothelial dysfunction to cognitive decline. The relationship between peripheral vascular function, measured by brachial artery FMD and RH, and AD dementia remains unclear. Few prospective studies have evaluated whether these measures are associated with AD risk and related brain changes over time.
2. **Interpretation**: This study found that lower peripheral vascular function was independently associated with a higher risk of developing AD dementia and cognitive impairment. These associations were stronger among individuals with evidence of systemic inflammation. Poor vascular health also correlated with adverse levels of plasma AD biomarkers, cerebrovascular pathologies, and brain atrophy. Although FMD is not a direct measure of endothelial function without pharmacological testing, it is partly mediated by endothelial‐dependent mechanisms, suggesting that peripheral vascular health may reflect aspects of brain microvascular and endothelial integrity relevant to AD risk.
3. **Future directions**: Further research is needed to evaluate the role of FMD and RH in predicting AD risk across diverse populations and to determine whether improving peripheral vascular function can mitigate AD risk. Interventional studies targeting vascular health through lifestyle, pharmacological, or anti‐inflammatory strategies may offer promising avenues for AD prevention.

### 2.2 Exposure measurement: endothelial function

FMD measurements were previously reported.^7^, ^21^ In brief, FMD was assessed using ultrasound imaging of the brachial artery with a 7.5‐MHz transducer. Arterial occlusion was induced for 5 min using a forearm cuff at a pressure of either 200 or 50 mmHg above systolic blood pressure. End‐diastolic images were digitally captured at baseline and for 2 min after deflation. Brachial artery diameter was measured offline by blinded sonographers using commercially available software (Brachial Analyzer, Medical Imaging Applications). FMD was calculated as both the absolute change in diameter (FMD mm) and the percentage change from baseline (FMD%). Blood flow velocity was measured by Doppler ultrasound and termed RH in centimeters per second (cm/s). Blinded personnel analyzed flow velocity at baseline and during FMD using a semiautomated signal‐averaging method.^22^

### 2.3 Primary outcomes

The primary outcomes included incident AD dementia, all‐cause dementia, and MCI. MCI and dementia were diagnosed by a review panel consisting of at least one neurologist and one neuropsychologist.^23^ FHS diagnostic criteria of dementia are based on the *Diagnostic and Statistical Manual of Mental Disorders*, Fourth Edition (DSM‐IV).^24^ The diagnosis of AD dementia was established based on the National Institute of Neurological and Communicative Disorders and Stroke (NINCDS) and the Alzheimer's Disease and Related Disorders Association (ADRDA).^25^, ^26^ All FHS participants were invited to have a cognitive assessment approximately every 5 years, with a subset of participants agreeing to it at each assessment cycle.^27^ Those aged >80 or who had been identified as cognitively impaired were invited for assessments on average around every 18 to 24 months. We diagnosed MCI using conventional Petersen–Winblad criteria (>1.5 standard deviations [SDs] below normal on one test within a cognitive domain) or comprehensive NP criteria developed by Jak et al. (>1 SD below normal on two tests within a domain).^28^

NP battery test domain scores were repeatedly assessed to capture cognitive trajectories across three domains: memory, executive function, and language. The detailed information was previously reported.^29^ In brief, each cognitive test item administered to participants was categorized into one of these domains based on expert consensus. Bifactor confirmatory factor analysis models for each domain were used to harmonize and calibrate cognitive data across multiple waves of the FHS exams. The data used in this study were longitudinal NP measures from participants who had at least two follow‐up NP measurements. NP testing occurred, on average, 7.4 ± 5.9 years after the baseline examination. We excluded participants who only had one NP battery test only before or after exam 7. Among the remaining participants after the exclusion, 500 had two waves, 670 had three waves, 401 had four waves, and 254 had five or more waves of NP battery testing, and their data were used. The number of NP waves was accounted for in the statistical models (described in the “Statistical Analysis” section). This methodology integrated item parameters from previously calibrated anchor items and estimated parameters for FHS‐specific items, facilitating the evaluation of cognitive trajectories over time.

### 2.4 Plasma AD biomarkers

We leveraged existing FHS data on plasma AD biomarkers to capture complementary aspects of amyloid and tau‐related neurodegeneration. These included plasma Aβ40 and Aβ42 measured at the baseline examination (offspring exam 7, concurrent with endothelial function assessments),^30^ as well as plasma t‐tau^31^ and p‐tau181^32^ measured at offspring exam 9 (mean 12.4 ± 0.8 years after baseline).

Plasma Aβ40 and Aβ42 were quantified using INNO‐BIA assays (Innogenetics, Ghent, Belgium), a multiplex microsphere‐based Luminex xMAP platform. Plasma t‐tau was measured using the Simoa™ Tau 2.0 Kit on the Simoa HD‐1 analyzer, and p‐tau181 was quantified using a highly sensitive Quanterix Single Molecule Array (Simoa) assay. Details regarding blood collection, processing, assay performance, and quality control procedures for plasma Aβ,^30^ t‐tau,^31^ and p‐tau181^32^ in the FHS were previously published. All biomarker values were log‐transformed to approximate normal distributions prior to analysis.

### 2.5 Brain MRI measurements

A subset of the FHS Gen 2 participants underwent brain MRI scanning between March 1999 and December 2017, as previously described,^33^ after the baseline measurements of FMD. Specifically, participants were imaged using a 1.5T MRI (Siemens Medical, Erlangen, Germany) with a three‐dimensional T1‐weighted coronal spoiled gradient‐recalled echo sequence. All images were transferred to and processed by the University of California Davis Medical Center without knowledge of the clinical information. Segmentation and quantification of the total cerebral cranial volume (TCV), frontal lobe brain volume (FBV), parietal lobe brain volume (PBV), temporal lobe brain volume (TBV), and hippocampal brain volume (HPV) were performed using semiautomated procedures as previously described. ^34^ TCV was determined using a convolutional neural network method.^35^ Non‐linear co‐registration of images to the Desikan–Killiany–Tourville atlas enabled the calculation of regional gray matter volumes.^36^ MRI measures were corrected for head size by calculating the percentage of these volumes relative to the TCV. The normalized brain volume variables were log‐transformed for normality. WMHs were segmented with fluid attenuated inversion recovery and gray matter by semi‐automated procedures, as previously described.^37^

The CMBs were defined as rounded or ovoid hypointense lesions on a T2*‐GRE weighted sequence, measuring <10 mm in diameter and surrounded by brain parenchyma over at least half the circumference of the lesion, followed recently published guidelines.^38^ The presence, number, and location of CMBs were determined. Reliability measures for CMB readings were previously described.^39^, ^40^

The MRI brain volumes, WMHs, and CMB data used in this study were after 1.4 ± 2.6 years, 7.7 ± 2.1 years, and 10.9 ± 6.9 years on average after the baseline exam, respectively. MRI brain volumes, WMH volume, and CMB prevalence were analyzed as secondary outcomes to evaluate the links with baseline FMD% and RH.

### 2.6 Statistical analysis

FHS participants were first divided into groups of those who (1) continued to experience normal cognition throughout the follow‐up period, (2) developed MCI, and (3) developed dementia during the follow‐up time. In descriptive statistics of all variables at baseline, ANOVA was used for continuous variables, and the chi‐squared test was used for categorical variables (Table 1, Table S1). Kaplan–Meier (KM) plots and log‐rank tests were used to test and compare the onsets of dementia and AD in tertiles of FMD% or RH.

**TABLE 1:** Baseline demographic characteristics, endothelial function, and medical conditions by cognitive status in Framingham Heart Study study sample.

Baseline characteristics	Total (*N* = 2844)	NC (*N* = 2325)	MCI (*N* = 241)	Dementia (*N* = 278)	*p* value
Age (year), mean (SD)	60.6 (9.4)	59.1 (8.99)	65.2 (7.68)	69.4 (7.67)	<0.001
Female, *n* (%)	1512 (53.2%)	1224 (52.6%)	125 (51.9%)	163 (58.6%)	0.15
Education, *n* (%)					<0.001
Less than high school	120 (4.2%)	78 (3.4%)	18 (7.5%)	24 (8.6%)	
High school graduate	874 (30.7%)	696 (29.9%)	87 (36.1%)	91 (32.7%)	
Some college	782 (27.5%)	628 (27.0%)	73 (30.3%)	81 (29.1%)	
College graduate	1050 (36.9%)	907 (39.0%)	63 (26.1%)	80 (28.8%)	
*APOE* genotypes					<0.001
*APOE* ε2	369 (13.0%)	307 (13.2%)	31 (12.9%)	31 (11.2%)	
*APOE* ε3	1788 (62.9%)	1500 (64.5%)	141 (58.5%)	147 (52.9%)	
*APOE* ε4	555 (19.5%)	414 (17.8%)	52 (21.6%)	89 (32.0%)	
Peripheral vascular measures, mean (SD)					
Baseline brachial diameter, mm	4.28 (0.868)	4.26 (0.86)	4.44 (0.91)	4.32 (0.86)	0.007
FMD, %	2.84 (2.76)	3.00 (2.84)	2.42 (2.46)	1.93 (2.12)	<0.001
Baseline mean flow, cm/s	8.19 (4.84)	8.37 (4.95)	7.74 (4.56)	6.97 (3.80)	<0.001
RH, cm/s	50.7 (21.3)	52.4 (21.4)	44.5 (19.2)	40.9 (18.8)	<0.001
Six‐min walk before FMD test, *n* (%)	1088 (38.3%)	908 (39.1%)	90 (37.3%)	90 (32.4%)	0.08
SBP (mmHg), mean (SD)	127 (18.8)	126 (18.7)	129 (18.2)	134 (18.8)	<0.001
DBP (mmHg), mean (SD)	74.2 (9.77)	74.5 (9.74)	73.0 (9.81)	72.1 (9.65)	<0.001
CVD, *n* (%)	352 (12.4%)	257 (11.1%)	40 (16.6%)	55 (19.8%)	<0.001
Hypertension, *n* (%)	1268 (44.6%)	973 (41.8%)	118 (49.0%)	177 (63.7%)	<0.001
Hypertension treatment, *n* (%)	936 (32.9%)	715 (30.8%)	91 (37.8%)	130 (46.8%)	<0.001
Diabetes, *n* (%)	308 (10.8%)	236 (10.2%)	27 (11.2%)	45 (16.2%)	0.010
Diabetes treatment, *n* (%)	182 (6.4%)	133 (5.7%)	19 (7.9%)	30 (10.8%)	0.003
Smoking, *n* (%)					0.003
Non‐smoker	1554 (54.6%)	1242 (53.4%)	140 (58.1%)	172 (61.9%)	
Former smoker	906 (31.9%)	744 (32.0%)	82 (34.0%)	80 (28.8%)	
Current smoker	384 (13.5%)	339 (14.6%)	19 (7.9%)	26 (9.4%)	
Lipid‐lowering medication, *n* (%)	588 (20.7%)	439 (18.9%)	62 (25.7%)	87 (31.3%)	<0.001
Heart rate (bpm), mean (SD)	65.0 (10.7)	64.9 (10.6)	65.0 (11.0)	65.9 (11.3)	0.36
Fasting glucose (mg/dL), mean (SD)	104 (26.1)	103 (25.3	106 (27.3)	109 (30.7)	0.002
Total cholesterol (mg/dL), mean (SD)	201 (36.8)	201 (36.9)	201 (35.8)	198 (37.6)	0.54
HDL (mg/dL), mean (SD)	53.7 (17.0)	53.8 (17.1)	53.5 (15.7)	52.7 (17.6)	0.56
Triglycerides (mg/dL), mean (SD)	137 (89.8)	137 (91.0)	137 (75.1)	142 (92.2)	0.65
BMI (kg/m2), mean (SD)	28.2 (5.31)	28.2 (5.36)	28.2 (5.28)	28.0 (4.94)	0.88
CRP ≥ 3 mg/dL (%)	1156 (40.6%)	932 (40.1%)	101 (41.9%)	123 (44.2%)	0.45

*Note*: *p* values were obtained using ANOVA for continuous variables and *χ* ^2^ tests for categorical variables.

Abbreviations: *APOE*, apolipprotein E; *APOE* ε2 includes ε2/ε2 and ε2/ε3; *APOE* ε3 includes ε3/ε3; *APOE* ε4 includes ε3/ε4 and ε4/ε4. *APOE* ε2/ε4 was not analyzed separately due to the opposing biological effects of the ε2 and ε4 alleles and its small sample size; BMI, body mass index; CRP, C‐reactive protein CVD, cardiovascular disease; DBP, diastolic blood pressure; FMD%, brachial artery flow‐mediated dilation; HDL, high‐density lipoprotein; MCI, mild cognitive impairment; NC, normal cognition; RH, reactive hyperemia; SBP, systolic blood pressure.

*Exposure modeling*: FMD% and RH were analyzed both as continuous variables and as tertiles to evaluate their associations with all primary outcomes, including incident AD, all‐cause dementia, MCI, plasma AD biomarkers, cognitive performance, and neuroimaging measures. Continuous modeling was used to preserve statistical power and to assess linear associations across the full distribution of vascular function. Tertile‐based analyses were prespecified to evaluate potential non‐linear relationships and to facilitate clinical interpretation by comparing participants with low, intermediate, and high vascular function.

*Time‐to‐event analysis (incident AD dementia, all‐cause dementia, and MCI)*: Cox proportional hazards models were used to estimate hazard ratios (HRs) and 95% confidence intervals (CIs) for the associations between baseline endothelial function measures FMD%, baseline mean flow, and RH and incident AD dementia, all‐cause dementia, and MCI. Participants were followed from baseline until event onset, death, loss to follow‐up, or end of follow‐up, whichever occurred first.

For the multivariable analyses, sensitivity analyses were conducted using two adjustment strategies (Table 2, Table S2). First, we applied a minimal adjustment set commonly used in dementia research, including baseline age, sex, and education. Second, covariate selection was further guided by a directed acyclic graph (DAG) informed by prior literature and domain expertise (Figure S2),^41^, ^42^ which identified a minimal sufficient adjustment set that incorporated apolipoprotein E (*APOE*) ε4 genotype and relevant cardiovascular risk factors. These DAG‐informed adjustments were prespecified as sensitivity analyses to assess the robustness of our findings.

**TABLE 2:** Associations of vascular function measures with risk of dementia and Alzheimer's disease.

					Without age cluster		With age cluster	
Outcome	Model	Predictor^a^	*n*	Event	HR [95% CI]	*p* value	HR [95% CI]	*p* value
Dementia	M1	FMD%	2585	276	0.78 [0.67, 0.90]	< 0.001	0.78 [0.69, 0.89]	< 0.001
		RH	2082	208	0.77 [0.65, 0.91]	0.002	0.77 [0.71, 0.83]	< 0.001
	M2	FMD%	2511	271	0.81 [0.69, 0.95]	0.009	0.81 [0.71, 0.93]	0.002
		RH	2018	205	0.89 [0.73, 1.07]	0.21	0.89 [0.78, 1.00]	0.052
Alzheimer's disease	M1	FMD%	2440	200	0.84 [0.71, 0.99]	0.045	0.83 [0.76, 0.91]	< 0.001
		RH	2026	152	0.79 [0.65, 0.96]	0.018	0.79 [0.75, 0.83]	< 0.001
	M2	FMD%	2440	200	0.83 [0.69, 0.996]	0.045	0.83 [0.76, 0.91]	< 0.001
		RH	1963	150	0.89 [0.71, 1.12]	0.32	0.89 [0.79, 0.999]	0.049

*Note*: Model 1 (M1): Adjusted for baseline age, sex, and education. Model 2 (M2): M1 plus additional adjustment for *APOE* ε4 status, walk test before FMD examination, systolic blood pressure, diastolic blood pressure, cardiovascular disease, hypertension, antihypertensive treatment, diabetes, diabetes treatment, smoking, lipid‐lowering treatment, high‐density lipoprotein, triglycerides, and fasting blood glucose. All models were first fitted without age‐cluster adjustment and then with age‐cluster adjustment.

Abbreviations: CI, confidence interval; FMD%, flow‐mediated dilation; HR, hazard ratio; RH, reactive hyperemia.

^a^Peripheral endothelial function measures (FMD% and RH) were standardized (*z*‐scores) from their raw values. Cox proportional hazards models were used to estimate HRs and 95% CIs for associations between vascular measures and incident all‐cause dementia or Alzheimer's disease.

To account for competing risk of death, cause‐specific hazard models were used, treating death as a competing event and censoring participants at their last observed follow‐up. Age‐cluster‐adjusted models were also examined as a sensitivity analysis to assess potential heterogeneity in dementia risk across different age periods. Effect modification was evaluated by testing interaction terms between each exposure (FMD% or RH) and sex, *APOE* ε4 status, and C‐reactive protein (CRP; ≥ 3 mg/dL vs < 3 mg/dL) in separate models. Stratified analyses were conducted for variables with statistically significant interactions (Table 3, Table S3).

**TABLE 3:** Interaction and stratified analyses of associations between peripheral vascular function measures and Alzheimer's disease risk.

		Dementia				Alzheimer's disease			
Interaction and strata	Predictor^a^	*n*	number of event	HR [95% CI]	*p* value	*n*	number of event	HR [95% CI]	*p* value
Interaction with sex	FMD% × Sex	2461	269	0.83 [0.76, 0.91]	< 0.001	2390	198	0.80 [0.72, 0.89]	< 0.001
	RH × Sex	1974	203	0.82 [0.67, 0.995]	0.045	1919	148	0.77 [0.57, 1.04]	0.09
Strata by sex									
Male	FMD%	1147	113	0.90 [0.81, 0.99]	0.038	1108	74	0.92 [0.87, 0.97]	0.001
	RH	903	85	0.90 [0.81, 0.996]	0.042	876	58	0.95 [0.86, 1.05]	0.33
Female	FMD%	1314	156	0.78 [0.64, 0.95]	0.015	1282	124	0.80 [0.70, 0.92]	0.002
	RH	1071	118	0.87 [0.70, 1.09]	0.23	1043	90	0.86 [0.67, 1.11]	0.24
Interaction with *APOE* status	FMD% × *APOE* ε4	2461	269	1.04 [0.67, 1.61]	0.86	2390	198	1.01 [0.59, 1.71]	0.98
	RH × *APOE* ε4	1974	203	1.29 [0.95, 1.75]	0.10	1919	148	1.19 [0.86, 1.64]	0.29
Strata by APOE status									
*APOE* ε4 non‐carriers	FMD%	1928	172	0.81 [0.65, 1.01]	0.06	1879	123	0.84 [0.68, 1.04]	0.11
	RH	1542	123	0.77 [0.69, 0.87]	< 0.001	1507	88	0.83 [0.79, 0.86]	<0.001
*APOE* ε4 carriers	FMD%	533	97	0.81 [0.59, 1.12]	0.20	511	75	0.82 [0.57, 1.19]	0.30
	RH	432	80	1.18 [0.97, 1.44]	0.11	412	60	1.10 [0.83, 1.46]	0.51
Interaction with CRP groups	FMD% × CRP	2461	269	0.74 [0.59, 0.91]	0.005	2390	198	0.75 [0.57, 0.97]	0.031
	RH × CRP	1974	203	0.73 [0.50, 1.07]	0.11	1919	148	0.74 [0.48, 1.15]	0.18
Strata by CRP group									
CRP < 3 mg/dL	FMD%	1458	150	0.93 [0.76, 1.14]	0.51	1418	110	0.97 [0.78, 1.20]	0.75
	RH	1167	116	1.14 [0.96, 1.35]	0.15	1136	85	1.17 [0.98, 1.40]	0.08
CRP ≥ 3 mg/dL	FMD%	1003	119	0.68 [0.64, 0.72]	< 0.001	972	88	0.70 [0.63, 0.79]	<0.001
	RH	807	87	0.63 [0.45, 0.89]	0.009	783	63	0.60 [0.39, 0.94]	0.026

*Note*: All interaction models were adjusted for baseline age, sex, education, *APOE* ε4 status, walk test before the FMD examination, systolic blood pressure, diastolic blood pressure, cardiovascular disease, hypertension, antihypertensive treatment, diabetes, diabetes treatment, smoking, lipid‐lowering treatment, high‐density lipoprotein, triglycerides, and fasting blood glucose, with age‐cluster adjustment. Stratified models were adjusted for the same covariates except for the stratification variable.

Abbreviations: CI, confidence interval; CRP, C‐reactive protein; FMD%, flow‐mediated dilation; HR, hazard ratio; RH, reactive hyperemia.

^a^ Peripheral endothelial function measures (FMD% and RH) were standardized (*z*‐scores) from their raw values. Cox proportional hazards models were used to estimate HRs and 95% CIs for associations between vascular measures and incident all‐cause dementia or Alzheimer's disease. Interaction analyses evaluated effect modification by sex, *APOE* ε4 status, and C‐reactive protein (CRP) (< 3 vs ≥3 mg/L). Stratified analyses were conducted by sex, *APOE* ε4 status, and CRP category.

*Longitudinal mixed‐effects models (cognitive performance outcomes)*: Linear mixed‐effects models were used to examine associations between endothelial function and longitudinal NP domain scores. Random intercepts were included to account for within‐participant correlations across repeated assessments. Models were adjusted for baseline age, sex, education, *APOE* ε4 status, walk test before the FMD exam, SBP, DBP, and CVD, hypertension, hypertension treatment, diabetes, diabetes treatment, smoking, lipid‐lowering treatment, HDL, triglycerides, and fasting blood glucose. For cognitive outcomes, FMD% and RH were modeled using tertiles to facilitate clinical interpretability and consistency with dementia risk analyses, given evidence of non‐linear associations in exploratory analyses.

Two model specifications were evaluated: Model 1 (M1) estimated baseline differences in cognitive performance (main effects only), and Model 2 (M2) additionally included interactions with follow‐up time to assess differences in cognitive change over time (Table 4). Sensitivity analyses were conducted using continuous measures of FMD% and RH (Table S4). Additional interaction and stratified analyses were performed to examine potential effect modification by sex, *APOE* ε4 genotype, and C‐reactive protein (CRP) levels (Tables S5 and S6).

**TABLE 4:** Associations between peripheral vascular function and cognitive domain performance.

Peripheral endothelial function	Models	Predictors (tertiles)	Memory		Executive function		Language	
			Beta [95% CI]	*p* value	Beta [95% CI]	*p* value	Beta [95% CI]	*p* value
FMD% (*n* = 1825)	M1	FMD% T1	Reference	–	Reference	–	Reference	–
		FMD% T2	−0.02 [−0.06, 0.03]	0.46	0.02 [−0.02, 0.07]	0.32	−0.01 [−0.06, 0.03]	0.60
		FMD% T3	0.00 [−0.05, 0.04]	0.83	0.05 [0.00, 0.09]	0.042	−0.02 [−0.07, 0.02]	0.34
	M2	FMD% T1 × time	Reference	–	Reference	–	Reference	–
		FMD% T2 × time	0.01 [−0.02, 0.04]	0.58	0.02 [−0.01, 0.06]	0.19	0.01 [−, 0.05]	0.55
		FMD% T3 × time	0.08 [0.04, 0.11]	< 0.001	0.09 [0.06, 0.13]	< 0.001	0.07 [0.03, 0.11]	< 0.001
RH (*n* = 1399)	M1	RH T1	Reference	–	Reference	–	Reference	–
		RH T2	0.02 [−0.03, 0.07]	0.44	0.02 [−0.03, 0.07]	0.44	0.00 [−0.05, 0.06]	0.97
		RH T3	0.01 [−0.04, 0.07]	0.67	0.05 [−0.01, 0.10]	0.12	0.01 [−0.05, 0.07]	0.77
	M2	RH T1 × time	Reference	–	Reference	–	Reference	–
		RH T2 × time	0.04 [0.00, 0.08]	0.029	0.07 [0.02, 0.11]	0.003	0.11 [0.07, 0.16]	< 0.001
		RH T3 × time	0.10 [0.06, 0.14]	< 0.001	0.11 [0.07, 0.15]	< 0.001	0.17 [0.12, 0.22]	< 0.001

*Note*: Participants were a subset with two or more NP assessments after baseline, when peripheral endothelial function was measured. Follow‐up time (Time) was defined as the interval from baseline to the date of the last neuropsychological assessment. Peripheral endothelial function measures (FMD% and RH) were categorized into tertiles (T1 to T3), with T1 as the reference group. Linear mixed‐effects models were used to examine associations of FMD% or RH with longitudinal cognitive performance in three domains (memory, executive function, and language).

All models were adjusted for baseline age, sex, education, *APOE* ε4 status, walk test before the FMD examination, systolic blood pressure, diastolic blood pressure, cardiovascular disease, hypertension, antihypertensive treatment, diabetes, diabetes treatment, smoking, lipid‐lowering treatment, high‐density lipoprotein, triglycerides, and fasting blood glucose. Model 1 (M1): Main effects only (baseline differences in cognitive performance). Model 2 (M2): Main effects plus interaction with time (differences in cognitive change over time).

Abbreviations: CI, confidence interval; FMD%, flow‐mediated dilation; RH, reactive hyperemia.

*Plasma AD biomarkers and neuroimaging associations*: Associations between endothelial function measures (FMD% and RH) and plasma AD biomarkers (Aβ measures and pTau181) or MRI‐derived brain measures were evaluated using non‐parametric methods (Kruskal–Wallis tests for continuous outcomes and *χ* ^2^ tests for CMB prevalence).

For these outcomes, which were assessed at a single time point and in smaller subsets of participants, both unadjusted models for all available variables and adjusted models for selected AD‐related variables were examined. Adjusted models included age at outcome assessment, sex, education, *APOE* ε4 genotype, and the time interval between endothelial function assessment and biomarker or MRI measurement. Minimally adjusted models were emphasized to preserve statistical power and reduce the risk of overadjustment; sensitivity analyses using expanded covariate sets were conducted where feasible and are reported in the supplementary materials. Unadjusted analyses were presented to illustrate the total (crude) associations between tertiles of peripheral vascular function and downstream AD‐related biological measures, which may share common vascular and inflammatory pathways. Corresponding adjusted results were provided to estimate associations independent of measured confounders for continuous variables. Effect modification by age was assessed for biomarker and neuroimaging outcomes by including interaction terms (age × FMD% and age × RH) (Tables S7–S9) as well as sex, *APOE* ε4 status, and C‐reactive protein (CRP; ≥ 3 mg/dL vs < 3 mg/dL) (Tables S10–S13).

### 2.7 General considerations

All analyses were conducted using R (version 4.4.0). Statistical significance was defined as a two‐sided *p* value < 0.05.

## 3 RESULTS

### 3.1 Comparisons of FMD test scores across cognitive controls, MCI and dementia

The 2844 individuals who completed the measurements of FMD% and RH and did not have MCI or dementia at the baseline were aged 60.6 ± 9.4 years old (mean ± SD), and 1512 (53.2%) were females (Figure S1). The average follow‐up years from the FMD measurement was approximately 17.5 ± 6.2 years. Participants were further categorized into three groups based on the outcomes at that time of follow‐up: (1) normal cognition (NC, *n* = 2325), (2) incident (MCI, *n* = 241), and (3) incident dementia (*n* = 278) (Table 1). Of the 278 individuals who developed dementia, 204 (73.4%) were diagnosed with AD. Compared to NC, the individuals who developed MCI and dementia were older, were more likely to be female, had lower education, and were more likely to carry *APOE* ε4 alleles. In addition, those who developed MCI and dementia were more likely to suffer from CVD but did not show differences in lipid status compared to the NC group.

Across the three groups, the NC group had the highest vasodilator function followed by MCI, and the dementia groups had the lowest levels of all vascular status measures (Table 1). For baseline brachial diameter, NC < MCI > dementia (mean ± SD: 4.26 ± 0.86 vs 4.44 ± 0.91 vs 4.32 ± 0.86, *p* = 0.007); for FMD% (mm), NC > MCI > dementia (mean ± SD: 3.00 ± 2.84 vs 2.42 ± 2.46 vs 1.93 ± 2.12, *p* < 0.001); for baseline mean flow, NC > MCI > dementia (mean ± SD: 8.37 ± 4.95 vs 7.74 ± 4.56 vs 6.97 ± 3.80, *p* < 0.001); and for RH, NC > MCI > dementia (mean ± SD: 52.4 ± 21.4 vs 44.5 ± 19.2 vs 40.9 ± 18.8, *p* < 0.001).

### 3.2 The associations between different vascular parameters and the risk of AD dementia

**FIGURE 1:**
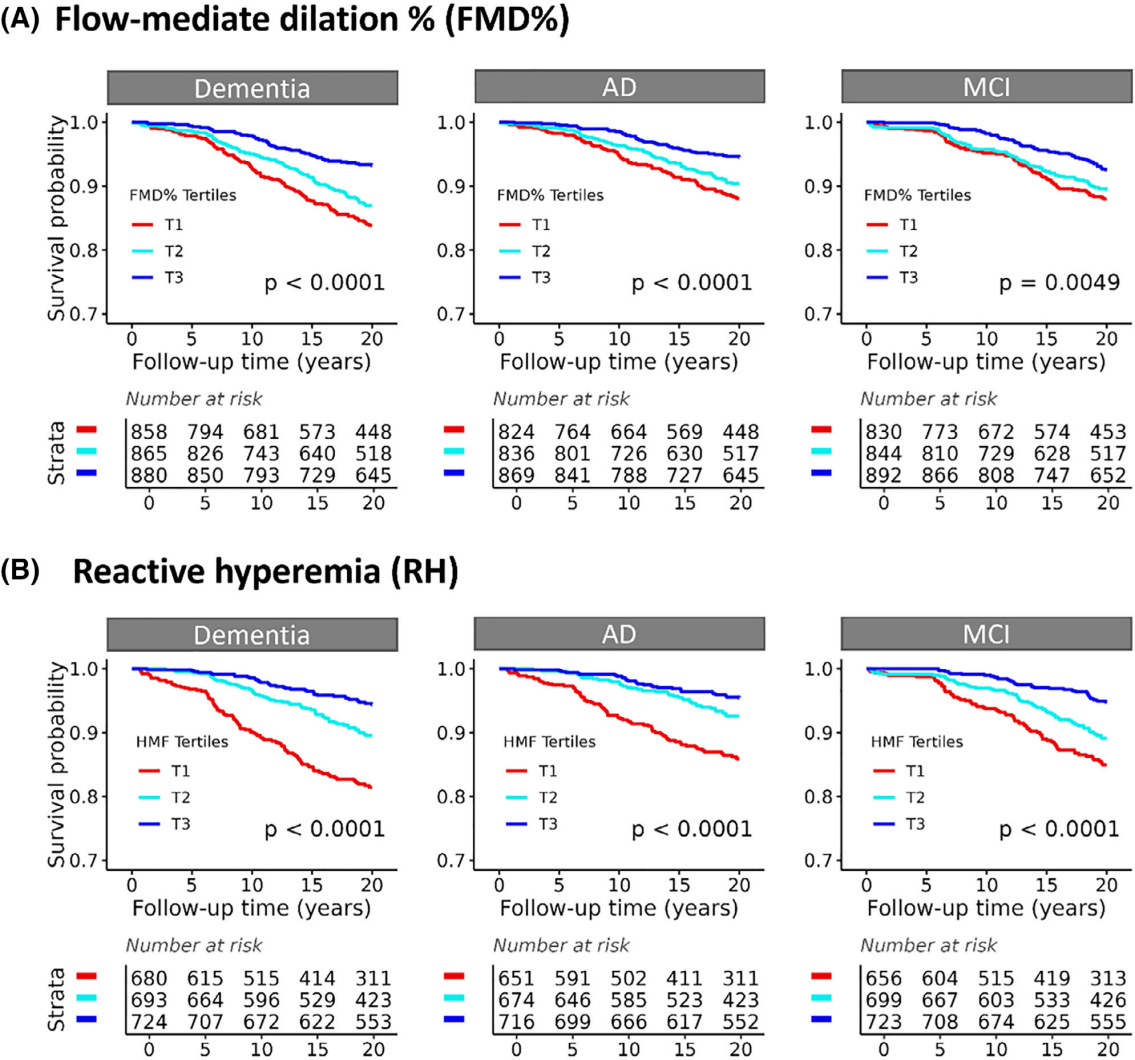
Kaplan–Meier analysis for free survival of dementia, Alzheimer's disease (AD) dementia, and mild cognitive impairment (MCI) in the tertiles of peripheral vascular function. Framingham Heart Study (FHS) participants were divided into three groups (T1, T2, and T3) based on the tertiles of peripheral vascular function, baseline brachial artery flow‐mediated dilation (FMD%) (A) and reactive hyperemia (RH) (B). Kaplan–Meier survival analysis was applied to survival free time before the onsets of dementia, AD, and MCI over 17 years of follow‐up. *p* values are shown.

To focus on the measurements of FMD% and RH, participants were first divided based on tertiles of FMD% (T1, T2, and T3) or RH (T1, T2, and T3). We conducted KM analyses and found that individuals with T1 of the FMD%‐poorest function (Figure 1A) had the highest rates and those with the T3‐highest function had the lowest rates of developing dementia (*p* < 0.0001), AD (*p* < 0.0001), and MCI (*p* = 0.0049) in a dose‐dependent manner. Similarly, in comparison, individuals with T1 of RH (Figure 1B) had the highest rates and those with T3 had the lowest rates of developing dementia (*p* < 0.0001), AD (*p* < 0.0001), and MCI (*p* < 0.0001). The results of T2 related to these risks fell in the middle. The characterizations across FMD% tertiles are shown in Table S1.

We then examined the continuous vascular variables FMD% and RH in relation to AD dementia risk using proportional hazards regression (Table 2). Two adjustment strategies were applied for sensitivity analyses. M1 covariate selection was guided by prior literature and included the minimal adjustment set commonly used in dementia research: age, sex, and education. M2 incorporated additional covariates identified by the DAG (Figure S2). In M1, for all‐cause dementia risk, FMD% showed HRs of 0.78 (95% CI: 0.67 to 0.90, *p* < 0.001) in the non‐age‐cluster model and 0.78 (95% CI: 0.69 to 0.89, *p* < 0.001) in the age‐cluster‐adjusted model; RH showed corresponding HRs of 0.77 (95% CI: 0.65 to 0.91, *p* = 0.002) and 0.77 (95% CI: 0.71 to 0.83, *p* < 0.001), respectively. Similarly, for AD risk, FMD% remained inversely related, with HRs of 0.84 (95% CI: 0.71 to 0.99, *p* = 0.045) in the non‐age‐cluster model and 0.83 (95% CI: 0.76 to 0.91, *p* < 0.001) in the age‐cluster‐adjusted model. RH showed comparable associations with AD risk, with HRs of 0.79 (95% CI: 0.65 to 0.96, *p* = 0.018) and 0.79 (95% CI: 0.75 to 0.83, *p* < 0.001), respectively. In M2, which further adjusted for *APOE* ɛ4 and cardiovascular risk factors, associations were slightly attenuated but remained directionally consistent (Table 2). For MCI, we found a positive relationship only between baseline brachial diameter and MCI (Table S2).

### 3.3 Stratifications of associations between different vascular parameters and risk of AD dementia

We then conducted proportional hazard regression analyses using interaction terms and stratifications. The interaction terms FMD% × Sex was associated with AD dementia, and stratification results showed that FMD% was negatively associated with AD dementia, with HR of 0.92 (95% CI 0.87 to 0.97, *p* = 0.001) in males and 0.80 (95% CI 0.70 to 0.92, *p* = 0.002) in females, respectively (Table 3). Similar associations were found for the relationship between FMD% and all‐cause dementia. In contrast, RH was only negatively associated with all‐cause dementia risk in males

The interaction terms FMD% × *APOE* ɛ4 and RH × *APOE* ɛ4 were not significantly associated with AD dementia. However, in stratifications, RH was negatively associated with all‐cause dementia and AD among *APOE* ɛ4 non‐carriers (Table 3).

While the interaction term FMD% × CRP 3 mg/dL was significant for both AD and all‐cause dementia, the RH × CRP 3 mg/dL interaction was not statistically significant, although it showed a similar trend. In stratified analyses, both FMD% and RH were negatively associated with all‐cause dementia and AD in the CRP ≥ 3 mg/L group, but not in the CPR < 3 mg/L group (Table 3). The stratified analyses for the MCI outcome had associations with FMD% and RH that were similar to those for AD dementia risk, especially in the presence of serum CRP ≥ 3 mg/L (Table S3).

### 3.4 Associations between different vascular parameters and cognitive decline

We examined the relationships between peripheral vascular function using tertiles of FMD% or RH and longitudinal domain‐specific cognitive performance (memory, executive function, and language domains), derived from harmonized NP summary scores collected across multiple waves. Linear mixed models (LMMs) were used to evaluate these associations. M1 estimated baseline differences in cognitive levels (main effects only), whereas M2 included interaction terms with follow‐up time to assess differences in cognitive change over time (Table 4). Specifically, M2 revealed significant associations between FMD% and cognitive function over time. Compared to T1, the highest tertile (T3) of FMD% was associated with a significant positive change in memory (FMD% T3 **×** Time: 0.08 [95% CI: 0.04, 0.11], *p* < 0.001), executive function (FMD% T3 **×** Time: 0.09 [95% CI: 0.06, 0.13], *p* < 0.001), and language (FMD% T3 **×** Time: 0.07 [95% CI: 0.03, 0.11], *p* < 0.001) over the follow‐up period (Table 4). The second tertile (T2) of FMD% interacted with time but did not show significant associations with changes in cognitive function. For RH, significant associations were also observed for cognitive trajectories over time. Compared to T1, both T2 and T3 of RH were significantly associated with positive changes in all cognitive domains (Table 4). The results using continuous exposures of FMD% and RH were consistent with the tertile‐based findings (Table S4). However, the interactive and stratification analyses of sex, *APOE* genotype, and CRP of FMD% and RH did not show statistical significance for cognitive domains (Tables S5 and S6).

### 3.5 Peripheral vascular function statuses associated with AD biomarkers and brain health

**FIGURE 2:**
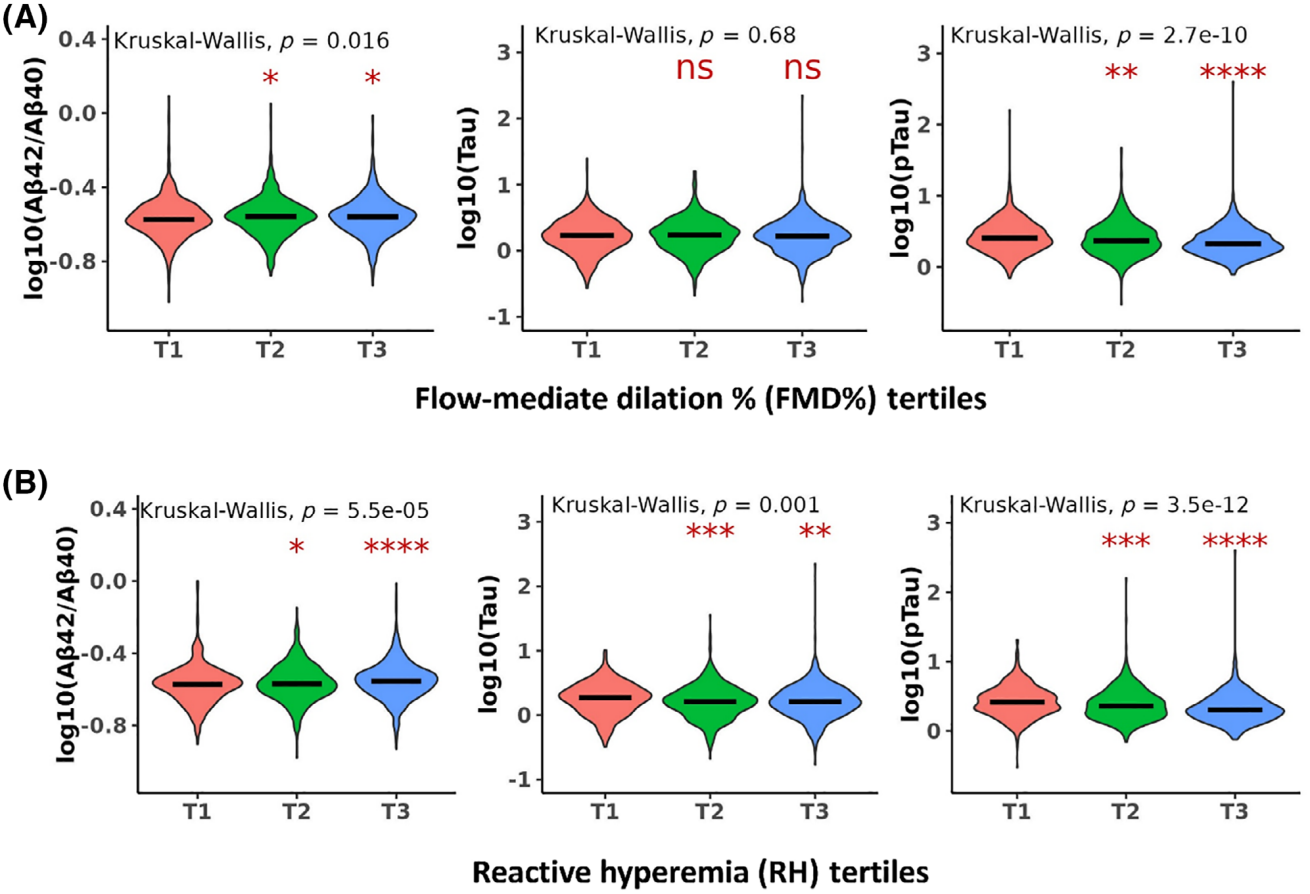
Comparisons of plasma Alzheimer's disease biomarkers based on the tertiles of peripheral vascular function. Framingham Heart Study (FHS) participants were divided into three groups (T1, T2, and T3) based on the tertiles of peripheral vascular function, baseline brachial artery flow‐mediated dilation (FMD%) (A) and reactive hyperemia (RH) (B). The *Z*‐scores of plasma amyloid beta (Aβ) 42/Aβ40 ratio, total tau and phosphorylated tau 181 (p‐tau181) were log‐transformed and the levels compared among three tertiles using Kruskal–Wallis analysis with overall statistical *p* values. To compare with T1 group, *p* values of either T2 or T3 for statistical significance are shown; *p < 0.05, **p < 0.01, ***p < 0.001, ****p < 0.0001.

We first examined plasma AD biomarkers across tertiles of FMD% and RH using Kruskal–Wallis tests. For FMD%, biomarker levels differed significantly across tertiles, with T3 (highest vascular function) showing the highest Aβ42/Aβ40 ratio (overall *p* = 0.016, T3 vs T2 *p* < 0.05) and the lowest level of plasma p‐tau181 (overall *p* = 2.7 × 10^−10^, T3 vs T1 *p* < 0.001) and T2 values generally falling between T1 and T3; t‐tau did not differ across FMD% tertiles (Figure 2A). Similarly, biomarker levels differed significantly across RH tertiles. T3 (healthiest RH) showed the highest Aβ42/Aβ40 ratio (overall *p* = 5.5 × 10^−5^, T3 vs T1 *p* < 0.001) and the lowest levels of plasma t‐tau (overall *p* = 0.001, T3 vs T1 *p* < 0.01) and p‐tau181 (overall *p* = 3.5 × 10^−12^, T3 vs T1 *p* < 0.001), and T2 values generally fell between T1 and T3 (Figure 2B).

**FIGURE 3:**
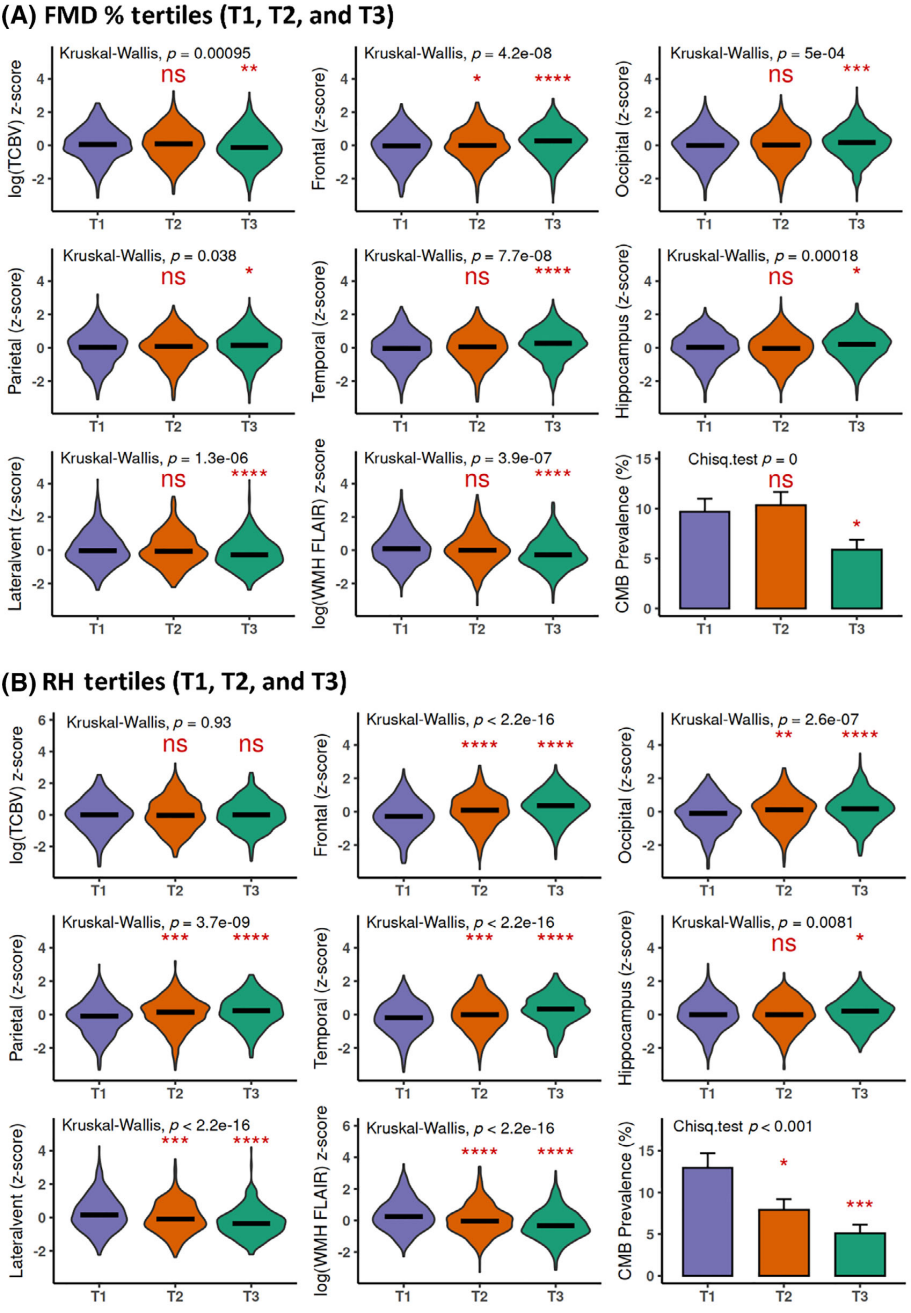
Comparisons of brain volumes and cerebrovascular pathologies based on the tertiles of peripheral vascular function. Framingham Heart Study (FHS) participants were divided into three groups (T1, T2, and T3) based on the tertiles of peripheral vascular function: baseline brachial artery flow‐mediated dilation percentage (FMD%) (A) and reactive hyperemia (RH) (B). The *Z*‐scores of brain volumes were log‐transformed and the levels compared among three tertiles using Kruskal–Wallis analysis with overall statistical *p* values. The absence and presence of CMBs were analyzed using chi‐squared (*χ* ^2^) tests with overall *p* values. To compare with T1 group, *p* values of either T2 or T3 for statistical significance are shown, *p < 0.05, **p < 0.01, ***p < 0.001, ****p < 0.0001. Brain volumes were measured by magnetic resonance imaging (MRI). CMB, cerebral microbleed; FBV, frontal lobe brain volume; HPV, hippocampal volume; PBV, parietal lobe brain volume; TBV, temporal lobe brain volume; TCBV,  total cerebral brain volume; WMH,  white matter hyperintensity.

Next, we examined the relationships between peripheral vascular function and MRI‐measured brain volumes using data from exams conducted after the baseline endothelial function assessments. Compared with participants with the poorest FMD% function (T1), those with the healthiest FMD% (T3) exhibited higher total cerebral brain volume (TCBV) volume, larger brain lobes (frontal, occipital, parietal, temporal), greater hippocampal volume, and lower lateral ventricle volume. Participants in T2 did not show statistically significant differences, except for a slight difference in the frontal lobe (Figure 3A). Compared to the poorest function of RH (T1), the participants with the healthiest RH (T3) had the highest volumes of all the brain lobes as well as the largest TCBV (Figure 3B). Compared to the measurements in T1 and T3, the brain measurements in T2, except TCBV and hippocampus, fell in the middle and reached statistical significance, suggesting a dose‐dependent relationship between peripheral vascular function and brain health.

We then studied brain vascular pathologies including WMHs (7.7 ± 2.1 years) and CMBs (10.9 ± 6.9 years), which were evaluated following the baseline endothelial function measurements. Significantly, the FMD T3 group had the lowest volume of WMHs (*p* < 0.0001) and the lowest rate of CMB (*p* < 0.05); similarly, the RH T3‐healthiest function group had the lowest volume of WMHs (*p* < 0.0001) and the lowest rate of CMB (*p* < 0.0001).

We further studied the continuous variables of FMD% and RH and their relationships with AD‐related markers, that is, plasma Aβ42/Aβ40, t‐tau, and p‐tau181, in addition to the outcomes related to cerebrovascular pathologies and AD pathology, that is, CMBs, WMHs, and hippocampal atrophy). After adjusting for age and other core covariates, the previously observed unadjusted associations between FMD%/RH and plasma AD biomarkers or MRI measures were attenuated and no longer statistically significant. Interaction tests (age × FMD% and age × RH) were statistically significant for several biomarkers (Tables S7–S9), indicating that the strength of associations varied across age. However, when we dichotomized age (<65 vs ≥65) and repeated stratified analyses, only the relationship between RH and Aβ42/40 ratio was significant in the group of age < 65 (β [95% CI]: 0.08 [0.02, 0.13], *p* = 0.005). Interaction and stratification analyses of sex, *APOE* genotypes, and CRP cutoff levels were not statistically significant for cross‐sectional blood biomarkers or MRI outcomes (Tables 10–13).

## 4 DISCUSSION

Emerging evidence implicates cerebrovascular dysfunction in the pathogenesis of AD, including BBB disruption and endothelial injury.^5^, ^6^ In this community‐based longitudinal study, we found that impaired peripheral vascular function in midlife, assessed by FMD% and RH, was associated with a higher risk of AD dementia and accelerated cognitive decline later in life. These associations were independent of traditional vascular risk factors and *APOE* genotype. Consistent with this, better endothelial function was associated with more favorable cognitive trajectories over time. Together, these findings extend prior cross‐sectional studies reporting lower FMD in individuals with AD or vascular dementia and its association with global cognitive performance^43^ by demonstrating a longitudinal link between midlife vascular function and late‐life cognitive outcomes.

This study found that lower FMD% and RH not only preceded clinical AD onset (Table 2) but also correlated with plasma biomarkers of AD pathology, including higher levels of p‐tau181 and lower Aβ42/Aβ40 ratios (Figure 2). These associations provide biological support for a mechanistic link between systemic vascular dysfunction including endothelia and AD‐related neurodegeneration. Histopathological studies show altered expression of adherens junction proteins (e.g., CD31, VE cadherin) in AD brains,^6^ with elevated tau species, including p‐tau217, present in cerebral microvessels.^13^, ^14^, ^44^ Our group previously demonstrated increased binding of monomeric C‐reactive protein (mCRP) to CD31 in AD, particularly in *APOE* ε4 carriers, leading to endothelial activation and p‐tau accumulation.^13^, ^14^ These findings support the vascular hypothesis of AD, indicating that systemic endothelial dysfunction may reflect parallel cerebrovascular injury, such as BBB breakdown, microvascular/endothelial inflammation, and impaired tau clearance, that precedes neurodegeneration in the brain.

We also found that lower FMD% and RH were negatively associated with greater WMHs and CMBs and reduced brain volumes (Figure 3). In contrast, the Cardiovascular Health Study, which examined an older cohort than the present study, did not observe statistically significant associations between FMD and incident AD.^11^ This discrepancy may reflect differences in the timing of vascular assessment and follow‐up time, as endothelial function measured in midlife may be more informative for subsequent brain injury and AD risk than assessments performed later in life, when competing risks, comorbidity burden, and selective survival may attenuate associations and limit the ability to detect effect modification, including by sex.^7^ It is widely recognized that CVDs increase the risk of AD and other dementias during aging.^45^, ^46^ Consistently, microvascular dysfunction and neurovascular uncoupling are exacerbated in peripheral artery disease, increasing the risk of cognitive decline in older adults.^47^ CMBs and WMHs are among such cerebrovascular abnormalities, defined as small chronic brain hemorrhages likely caused by structural abnormalities, including the endothelia of the brain.^48^, ^49^, ^50^ CMBs have been associated with cognitive impairment and increased risk for AD development across multiple studies.^51^, ^52^ As endothelial dysfunction leads to or co‐exists with CMBs and WMHs, we observed sex differences in the vascular–AD relationship. The stronger FMD–AD link in females may reflect a loss of estrogen's endothelial protection after menopause,^50^ while microvascular impairment (RH) may be more relevant in males, who develop earlier small vessel disease.^53^ We noticed that the attenuation of AD biomarker and neuroimaging associations occurred after using the continuous variables of FMD and RH and covariate adjustment (Figures 2 and 3; Tables S10–S13). This underscores the complexity of linking systemic vascular function to molecular and structural brain changes and highlights that FMD and RH may reflect integrative physiological processes that are more closely aligned with clinical outcomes than with individual biomarker measures. On the other hand, plasma biomarkers and MRI measures were assessed at a single time point, which limits effective sample size, increases susceptibility to interindividual variability, and substantially reduces power for detecting interaction terms, particularly those of modest effect size. Interaction tests are inherently less powered than main effects, and this limitation is amplified for cross‐sectional continuous outcomes with higher measurement variability.

Notably, the association between impaired endothelial function and AD risk was stronger among individuals with elevated systemic inflammation (CRP ≥ 3 mg/L), supporting the hypothesis that peripheral inflammation amplifies cerebrovascular vulnerability, particularly in genetically at‐risk populations. It has been shown that mCRP causes endothelial inflammation and damage, leading to neuroinflammation and AD pathology, especially in the brains of individuals carrying the *APOE* ε4 risk allele.^14^, ^54^, ^55^, ^56^ In the neurodegenerative brain, endothelial cells exhibit activation of complements and coagulation pathways, leading to endothelial junction damage and neuroinflammation.^1^, ^5^, ^57^, ^58^ The endothelial expression of adherens junctions is significantly decreased in the AD brain.^14^, ^59^, ^60^ Additionally, the endothelium plays roles in preventing amyloid accumulation and neuroinflammation in the AD brain.^61^, ^62^

FMD% and RH provide complementary insights into endothelial health: FMD primarily reflects nitric oxide (NO)‐mediated conduit artery function,^63^ while RH captures microvascular reactivity and resistance vessel adaptation.^8^, ^64^, ^65^ FMD measures vasodilation of conduit arteries in response to increased shear stress, a response largely mediated by endothelial NO release, making it a well‐validated indicator of endothelial function in vivo.^66^, ^67^ Although these measures are derived from peripheral circulation, prior studies and our data have shown associations between brachial FMD and cerebral vascular function status.^9^, ^10^ Impaired NO signaling and endothelial activation may compromise BBB integrity, promote hypoperfusion, and facilitate perivascular tau accumulation, which are the mechanistic pathways increasingly recognized in AD. Although age × FMD/RH interactions indicate age‐dependent variation, the lack of consistent findings in dichotomized age groups suggests no clear age threshold. Overall, by demonstrating that both FMD and RH impairments precede cognitive decline and AD biomarker elevation, our study provides vascular‐level evidence to warrant larger studies with continuous age modeling to define when these relationships are strongest during the life span.

This study has several limitations. First, because we did not perform nitroglycerin‐mediated dilation, we cannot exclude contributions from vascular smooth muscle and other non‐endothelial factors for FMD.^67^ Second, the FMD assessments were conducted between 1998 and 2001 and did not follow the standardized protocols for FMD, before the standardized protocols were published.^68^ Third, lack of repeated FMD and RH measurements over time precludes evaluation of longitudinal changes in vascular function. These limitations may partly explain the observed interaction effects of sex, *APOE* genotypes, and CRP cutoff levels for incident AD and dementia, but not for MCI, or for cross‐sectional blood biomarkers or MRI outcomes. Fourth, while FMD and RH are widely used peripheral vascular markers, they are not direct measures of cerebrovascular function. Fifth, since only a subset of FHS participants agreed to a cognitive assessment at each test cycle, the transition phase of MCI between normal cognition and dementia may not have been fully captured. Finally, the predominantly White study population limits generalizability to other racial and ethnic groups.

In conclusion, midlife peripheral vascular and endothelial dysfunction, as indicated by impaired FMD% and RH, was associated with later‐life risk of AD dementia, cognitive decline, and cerebral small vessel disease. These findings underscore the potential utility of non‐invasive peripheral vascular and endothelial biomarkers for early identification of individuals at heightened risk for AD. Moreover, they support growing interest in targeting vascular–endothelial pathways in the prevention and treatment of AD, particularly in the context of systemic inflammation and *APOE* genotype. Future studies should test whether interventions known to improve endothelial function, such as aerobic exercise, statins, or anti‐inflammatory therapies, also reduce AD biomarker progression or cognitive decline. Trials incorporating FMD/RH as a surrogate endpoint could clarify its utility as a preventive biomarker for AD.

## CONFLICT OF INTEREST STATEMENT

The authors declare no conflicts of interest related to this work. Outside the submitted work, Rhoda Au serves as a scientific advisor to Signant Health and NovoNordisk. Author disclosures are available in the Supporting Information.

## CONSENT STATEMENT

The study was approved by the IRB of the Boston University School of Medicine and conducted in accordance with the ethical standards of the 1964 Declaration of Helsinki and its later amendments or comparable ethical standards. All participants provided written informed consent prior to participation.

## Supporting information



Supporting Information

Supporting Information

## Data Availability

The data used in this study are available upon request from the FHS through a formal data request process (https://www.framinghamheartstudy.org/fhs‐for‐researchers/research‐application/). Researchers may access the data by submitting a research proposal to the FHS, subject to review and approval in accordance with FHS data access policies.
