# Supplementary material for: Peripheral vascular function, including endothelium‐dependent measures, and dementia risk: The Framingham Heart Study
Source: Alzheimers Dement. 2026 Apr 28;22(5):e71396. doi: 10.1002/alz.71396 (PMC13124649; doi:10.1002/alz.71396)
Supplement: Supplementary file 2 — Supporting Information [file ALZ-22-e71396-s001.docx]

# Supplementary Figures and Tables


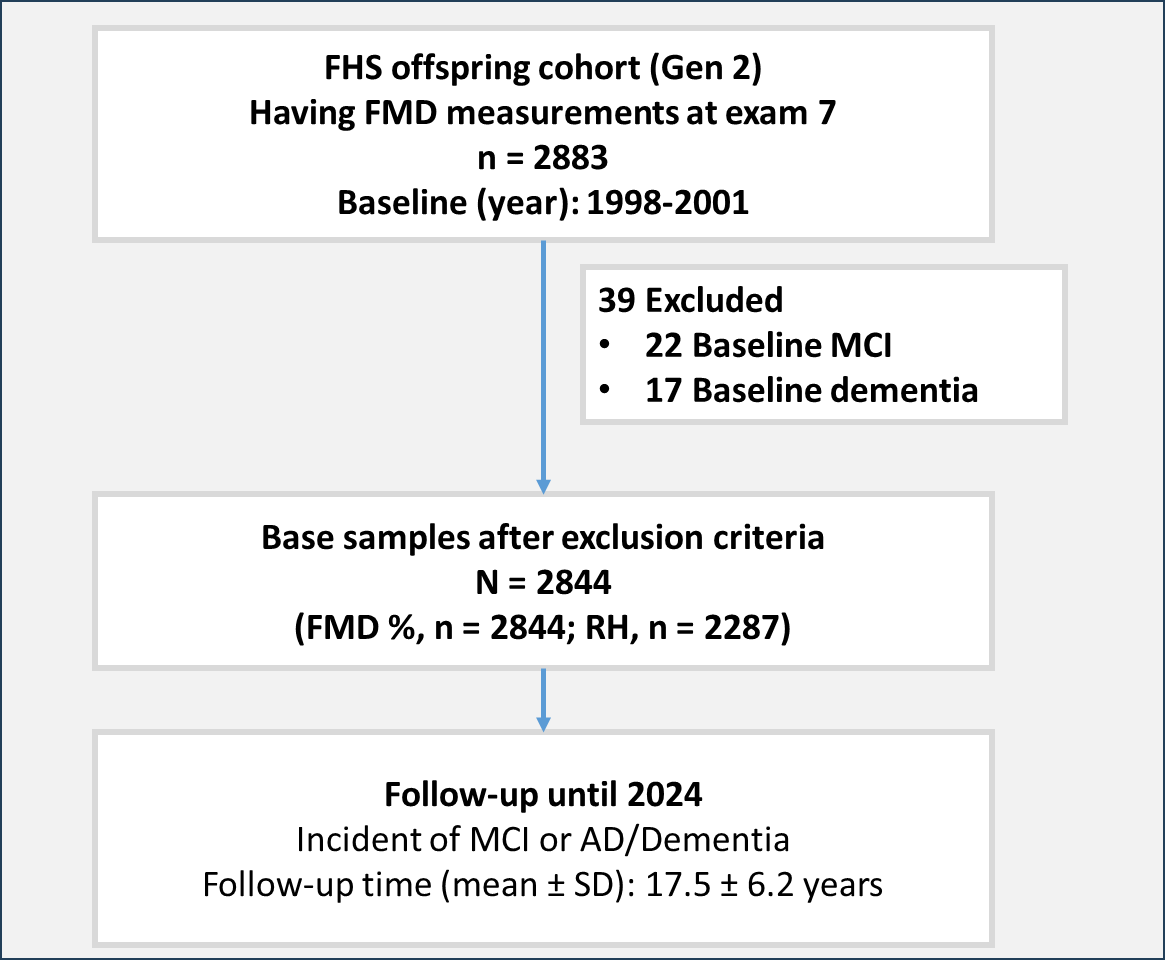


**Supplementary Figure S1**. Flow-chart of the selection of study subjects

This study included participants from the Framingham Heart Study (FHS) Offspring Cohort (Gen 2) who had flow-mediated dilation (FMD) measurements at Exam 7 (1998–2001). Of the 2,883 participants initially assessed, 39 were excluded due to baseline mild cognitive impairment (MCI, n = 22) or dementia (n = 17), resulting in a final analytic sample of 2,844 individuals (FMD %, n = 2,844; reactive hyperemia [RH], n = 2,287). Participants were followed up through 2024 to evaluate the incidence of MCI or Alzheimer’s disease (AD)/dementia, with a mean follow-up time of 17.5 ± 6.2 years.


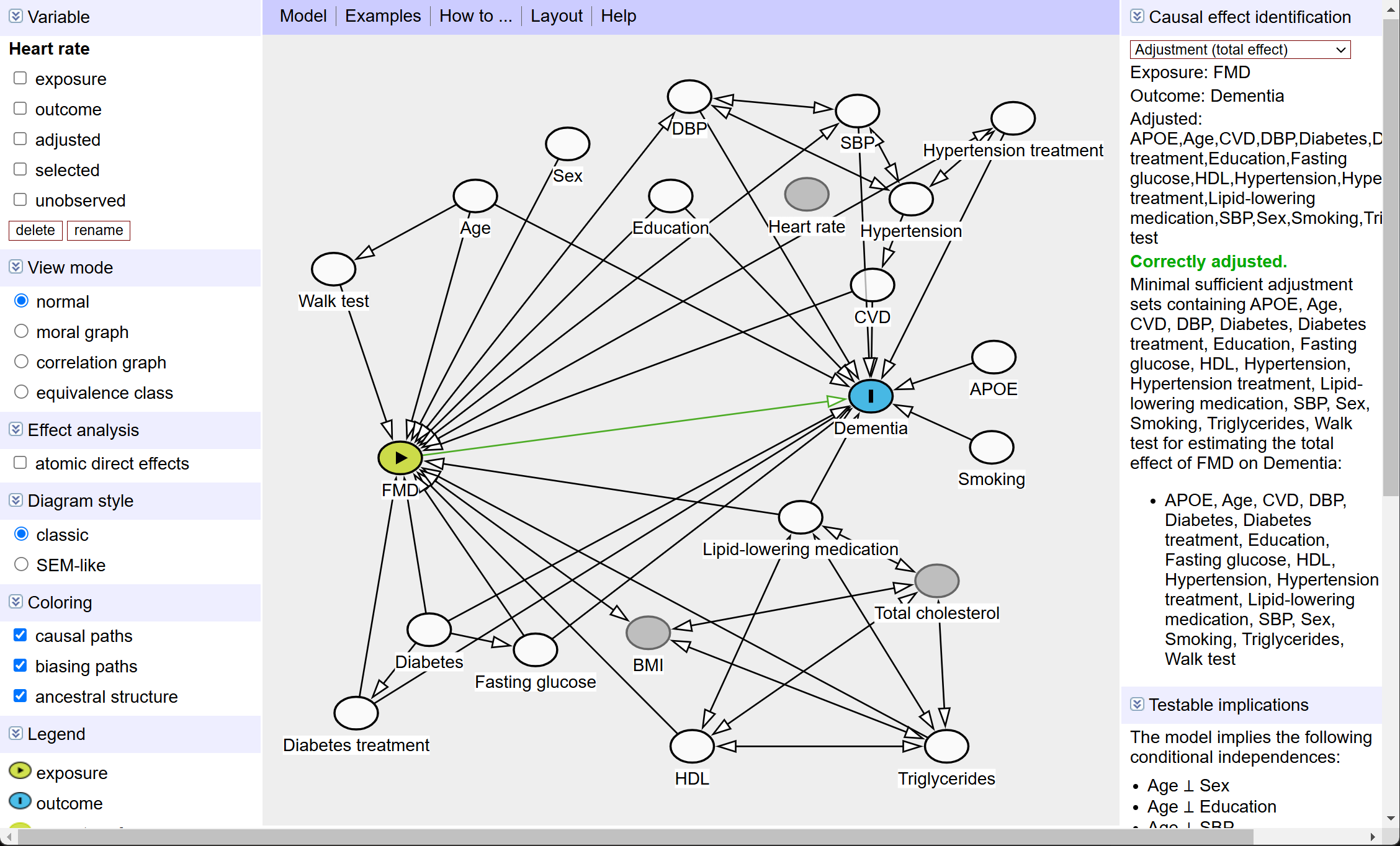


**Supplementary Figure S2**. Directed Acyclic Graph (DAG) Illustrating Minimal Sufficient Adjustment for Causal Analysis.

This DAG depicts the hypothesized causal structure between flow-mediated dilation (FMD; exposure) and dementia (outcome) based on observed covariates in the Framingham Heart Study. Arrows represent assumed causal relationships between variables. The DAG identifies a minimal sufficient adjustment set to estimate the total effect of FMD on dementia risk, as determined using DAGitty (https://www.dagitty.net/). Variables in this adjustment set include APOE, age, cardiovascular disease (CVD), diastolic blood pressure (DBP), diabetes, diabetes treatment, education, fasting glucose, HDL cholesterol, hypertension, hypertension treatment, lipid-lowering medication, systolic blood pressure (SBP), sex, smoking, triglycerides, and walk test. Adjusting for these covariates blocks all non-causal (backdoor) paths from FMD to dementia, allowing for unbiased causal inference. The green arrow indicates the direct effect of FMD on dementia.

**Supplementary Table S1: Clinical measures among tertile groups of FMD%**

| **Baseline characteristics** | **FMD% T1**  **[-3.32, 1.34)**  **(N=949)** | **FMD% T2**  **[ 1.34, 3.56)**  **(N=947)** | **FMD% T3**  **[ 3.56,17.97]**  **(N=948)** | **P-value** |
| --- | --- | --- | --- | --- |
| **Age (year), Mean ± SD** | 63.0 ± 9.02 | 61.6 ± 9.46 | 57.2 ± 8.66 | <0.001 |
| **Female, n (%)** | 435 (45.8%) | 484 (51.1%) | 593 (62.6%) | <0.001 |
| **Education, n (%)** |  |  |  | 0.06 |
| Less than high school | 50 (5.3%) | 45 (4.8%) | 25 (2.6%) |  |
| High school graduate | 300 (31.6%) | 283 (29.9%) | 291 (30.7%) |  |
| Some college | 245 (25.8%) | 255 (26.9%) | 282 (29.7%) |  |
| College graduate | 347 (36.6%) | 358 (37.8%) | 345 (36.4%) |  |
| **APOE Genotypes** |  |  |  | <0.001 |
| APOE2 | 127 (13.4%) | 112 (11.8%) | 130 (13.7%) |  |
| APOE3 | 600 (63.2%) | 610 (64.4%) | 578 (61.0%) |  |
| APOE4 | 181 (19.1%) | 174 (18.4%) | 200 (21.1%) |  |
| **Peripheral endothelial function,** Mean ± SD |  |  |  |  |
| Baseline brachial diameter, mm | 4.54 ± 0.898 | 4.34 ± 0.81 | 3.97 ± 0.80 | <0.001 |
| Flow-mediate dilation, % | 0.14 ± 0.88 | 2.40 ± 0.62 | 5.99 ± 2.08 | <0.001 |
| Baseline mean flow, cm/S | 7.81 ± 4.46 | 7.61 ± 4.63 | 9.18 ± 5.26 | <0.001 |
| Reactive hyperemia (RH), cm/s | 39.0 ± 16.7 | 49.7 ± 19.6 | 63.9 ± 19.7 | <0.001 |
| **Six minutes walk before FMD test, n (%)** | 311 (32.8%) | 339 (35.8%) | 438 (46.2%) | <0.001 |
| **SBP (mmHg), Mean ± SD** | 133 ± 19.7 | 127 ± 18.6 | 120 ± 15.5 | <0.001 |
| **DBP (mmHg), Mean ± SD** | 75.4 ± 10.5 | 73.9 ± 9.78 | 73.1 ± 8.88 | <0.001 |
| **CVD, n (%)** | 158 (16.6%) | 129 (13.6%) | 65 (6.9%) | <0.001 |
| **Hypertension, n (%)** | 560 (59.0%) | 423 (44.7%) | 285 (30.1%) | <0.001 |
| **Hypertension treatment, n (%)** | 399 (42.0%) | 324 (34.2%) | 213 (22.5%) | <0.001 |
| **Diabetes, n (%)** | 144 (15.2%) | 96 (10.1%) | 68 (7.2%) | <0.001 |
| **Diabetes treatment, n (%)** | 85 (9.0%) | 56 (5.9%) | 41 (4.3%) | <0.001 |
| **Smoking, n (%)** |  |  |  | 0.36 |
| Nonsmoker | 517 (54.5%) | 537 (56.7%) | 500 (52.7%) |  |
| Quit smoker | 306 (32.2%) | 294 (31.0%) | 306 (32.3%) |  |
| Current smoker | 126 (13.3%) | 116 (12.2%) | 142 (15.0%) |  |
| **Lipid-lowering medication, n (%)** | 251 (26.4%) | 208 (22.0%) | 129 (13.6%) | <0.001 |
| **Heart rate (bpm), Mean ± SD** | 65.2 ± 11.4 | 63.9 ± 10.3 | 65.9 ± 10.3 | <0.001 |
| **Fasting glucose (mg/dL), Mean ± SD** | 107 ± 29.0 | 105 ± 25.9 | 101 ± 22.7 | <0.001 |
| **Total cholesterol (mg/dL), Mean ± SD** | 199 ± 38.3 | 200 ± 35.8 | 202 ± 36.3 | <0.001 |
| **HDL (mg/dL), Mean ± SD** | 51.6 ± 16.4 | 53.9 ± 17.4 | 55.6 ± 17.0 | 0.18 |
| **Triglycerides (mg/dL), Mean ± SD** | 145 ± 91.8 | 133 ± 85.6 | 133 ± 91.6 | 0.004 |
| **BMI (kg/m2), Mean ± SD** | 28.8 ± 5.59 | 28.0 ± 5.08 | 27.6 ± 5.18 | <0.001 |
| **CRP ≥ 3 mg/dL (%)** | 403 (42.5%) | 388 (41.0%) | 365 (38.5%) | 0.22 |

The p-values (two-tailed tests) were obtained using ANOVA tests for continuous variables and Chi-square (*χ*²) tests for categorical variables.

**Supplementary Table S2.** Relation of vascular function measures and risk of mild cognitive impairment (MCI)

| **Outcome** | **Model** | **Predictor ***  **(Peripheral endothelial function)** | **n** | **nevent** | **No age cluster adjusting** | |  | **Age cluster adjusting** | |
| --- | --- | --- | --- | --- | --- | --- | --- | --- | --- |
|  |  |  |  |  | **HR [95CI%]** | **P values** |  | **HR [95CI%]** | **P values** |
| **MCI** | **M1** | Baseline brachial diameter, mm | 2550 | 241 | 1.27 [1.06, 1.52] | 0.009 |  | 1.27 [1.11, 1.45] | < 0.001 |
|  |  | FMD % | 2550 | 241 | 0.94 [0.81, 1.08] | 0.40 |  | 0.94 [0.85, 1.04] | 0.23 |
|  |  | Baseline mean flow, cm/S | 2064 | 190 | 1.01 [0.86, 1.18] | 0.89 |  | 1.01 [0.82, 1.24] | 0.92 |
|  |  | RH, cm/s | 2064 | 190 | 0.86 [0.73, 1.02] | 0.08 |  | 0.86 [0.72, 1.03] | 0.10 |
|  | **M2** | Baseline brachial diameter, mm | 2418 | 226 | 1.23 [1.02, 1.49] | 0.03 |  | 1.23 [1.04, 1.46] | 0.016 |
|  |  | FMD % | 2418 | 226 | 0.93 [0.80, 1.09] | 0.38 |  | 0.93 [0.83, 1.05] | 0.24 |
|  |  | Baseline mean flow, cm/s | 1949 | 178 | 0.99 [0.83, 1.17] | 0.87 |  | 0.99 [0.83, 1.17] | 0.87 |
|  |  | RH, cm/s | 1949 | 178 | 0.86 [0.71, 1.04] | 0.12 |  | 0.86 [0.69, 1.06] | 0.16 |

Cox proportional hazard models were employed to estimate hazard ratios (HRs) and 95% confidence intervals (CIs) for the associations between vascular measures and incident MCI. *All predictors (peripheral vascular measures including endothelial function) were transformed into z-scores from their raw values.

Model 1 (M1): Adjusted for baseline age, sex, and education.

Model 2 (M2): M1 plus adjusted APOE4, walk test before FMD exam, SBP, DBP, CVD, hypertension, hypertension treatments, diabetes, diabetes treatments, smoking, lipid treatments, HDL, triglycerides, and fasting blood glucose.

All models were first fitted without age cluster adjustment and then with age cluster adjustment.

Abbreviations: FMD % = Brachial artery flow-mediated dilation (FMD %); RH = Reactive hyperemia, i.e. hyperemic mean flow during FMD test.

**Supplementary Table S3. Stratified Analysis of MCI Onset Based on Five Peripheral Endothelial Function Measurements**

| **Interaction**  **and Strata** | | **Predictor ***  **(Peripheral endothelial function)** | **MCI** | | | |
| --- | --- | --- | --- | --- | --- | --- |
|  |  |  | **n** | **nevent** | **HR [95CI%]** | **P values** |
| **Interaction: Sex** | | FMD % **×** Sex | 2418 | 226 | 1.01 [0.86, 1.17] | 0.94 |
|  |  | RH, cm/s **×** Sex | 1949 | 178 | 1.43 [1.18, 1.72] | < 0.001 |
| **Strata:**  **sex** | **Male** | FMD % | 1143 | 109 | 0.99 [0.83, 1.18] | 0.91 |
|  |  | RH, cm/s | 898 | 80 | 0.72 [0.56, 0.93] | 0.011 |
|  | **Female** | FMD % | 1275 | 117 | 0.90 [0.83, 0.98] | 0.019 |
|  |  | RH, cm/s | 1051 | 98 | 1.00 [0.80, 1.24] | 0.98 |
| **Interaction: APOE4** | | FMD % **×** APOE ε4 | 2418 | 226 | 0.86 [0.73, 1.03] | 0.11 |
|  |  | RH, cm/s **×** APOE ε4 | 1949 | 178 | 0.99 [0.61, 1.59] | 0.96 |
| **Strata:**  **APOE4** | **ε4 (-)** | FMD % | 1924 | 168 | 0.99 [0.84, 1.17] | 0.93 |
|  |  | RH, cm/s | 1549 | 130 | 0.85 [0.62, 1.17] | 0.31 |
|  | **ε4 (+)** | FMD % | 494 | 58 | 0.81 [0.64, 1.02] | 0.08 |
|  |  | RH, cm/s | 400 | 48 | 0.94 [0.51, 1.75] | 0.85 |
| **Interaction: CRP (≥ 3 vs <3)** | | FMD % **×** CRP 3 mg/dL | 2418 | 226 | 0.86 [0.67, 1.12] | 0.27 |
|  |  | RH, cm/s **×** CRP 3 mg/dL | 1949 | 178 | 0.94 [0.80, 1.11] | 0.48 |
| **Strata:**  **CRP**  **(mg/dL)** | **< 3** | FMD % | 1438 | 130 | 1.00 [0.83, 1.22] | 0.98 |
|  |  | RH, cm/s | 1151 | 100 | 0.93 [0.74, 1.16] | 0.51 |
|  | **≥ 3** | FMD % | 980 | 96 | 0.84 [0.76, 0.93] | < 0.001 |
|  |  | RH, cm/s | 798 | 78 | 0.78 [0.63, 0.95] | 0.014 |

Cox proportional hazards models were used to estimate hazard ratios (HRs) and 95% confidence intervals (CIs) for the associations between vascular measures and incident MCI, including analyses by strata and interaction terms. All predictors (peripheral endothelial function measures) were transformed into z-scores. All models were adjusted for baseline age, sex (in APOE strata only), education, APOE4 (in sex strata only), walk test prior to the FMD exam, SBP, DBP, CVD, hypertension, hypertension treatment, diabetes, diabetes treatment, smoking, lipid-lowering treatment, HDL, triglycerides, and fasting blood glucose, with age-cluster adjustment applied where appropriate.

**Supplementary Table S4. The relationship between peripheral vascular function and cognitive function domains**

| **Peripheral**  **Endothelial**  **function** | **Models** | **Predictors** | **Memory** | |  | **Executive Function** | |  | **Language** | |
| --- | --- | --- | --- | --- | --- | --- | --- | --- | --- | --- |
|  |  |  | **Beta [95%CI]** | **P value** |  | **Beta [95%CI]** | **P value** |  | **Beta [95%CI]** | **P value** |
| **FMD %**  **(n=1825)** | **M1** | **FMD %** | -0.00 [-0.02, 0.01] | 0.71 |  | 0.01 [-0.01, 0.03] | 0.18 |  | -0.02 [-0.04, 0.00] | 0.053 |
|  | **M2** | **FMD %** | -0.03 [-0.05, -0.01] | 0.007 |  | -0.01 [-0.03, 0.01] | 0.23 |  | -0.06 [-0.11, -0.02] | 0.003 |
|  |  | **Time** | -0.14 [-0.15, -0.12] | < 0.001 |  | -0.24 [-0.25, -0.22] | < 0.001 |  | -0.02 [-0.06, 0.03] | 0.42 |
|  |  | **FMD% × Time** | 0.04 [0.02, 0.05] | < 0.001 |  | 0.04 [0.02, 0.05] | < 0.001 |  | 0.05 [0.03, 0.06] | < 0.001 |
| **RH**  **(n=1399)** | **M1** | **FMD %** | 0.01 [-0.01, 0.03] | 0.39 |  | 0.02 [0.00, 0.05] | 0.049 |  | 0.01 [-0.02, 0.03] | 0.69 |
|  | **M2** | **FMD %** | -0.02 [-0.05, 0.00] | 0.061 |  | -0.01 [-0.03, 0.02] | 0.6 |  | -0.08 [-0.13, -0.03] | 0.002 |
|  |  | **Time** | -0.13 [-0.15, -0.12] | < 0.001 |  | -0.25 [-0.27, -0.23] | < 0.001 |  | -0.02 [-0.07, 0.03] | 0.35 |
|  |  | **RH × Time** | 0.05 [0.03, 0.06] | < 0.001 |  | 0.04 [0.03, 0.06] | < 0.001 |  | 0.07 [0.05, 0.09] | < 0.001 |

Follow-up time ("Time") was defined as the interval from baseline (Exam 7; assessment of peripheral endothelial function) to the last neuropsychological (NP) test. Participants with no NP testing or only one NP test on or after baseline were excluded. Generalized linear mixed models (GLMMs) were fitted using the lme4 and lmerTest R packages. Scaled (z-score) flow-mediated dilation (FMD%) and reactive hyperemia (RH) were analyzed as continuous predictors of cognitive performance across three domains (memory, executive function, and language). Models were adjusted for baseline age, sex, education, APOE4 status, walk test prior to the FMD exam, SBP, DBP, CVD, hypertension and treatment, diabetes and treatment, smoking, lipid treatment, HDL, triglycerides, and fasting glucose. Model M1 included main effects only (baseline cognitive differences). Model M2 included main effects plus interaction with Time (cognitive change over time).

**Supplementary Table S5. Stratified Analysis of Cognitive function domains and FMD%**

| Models | Predictor | Memory | | |  | Executive Function | | |  | Language | | |
| --- | --- | --- | --- | --- | --- | --- | --- | --- | --- | --- | --- | --- |
|  |  | **N** | **Beta [95% CI]** | **P value** |  | **N** | **Beta [95% CI]** | **P value** |  | **N** | **Beta [95% CI]** | **P value** |
| A1 | **FMD%** | 1845 | -0.02 [-0.08, 0.04] | 0.6 |  | 1843 | 0.06 [-0.01, 0.12] | 0.091 |  | 1790 | -0.04 [-0.11, 0.02] | 0.19 |
|  | **Sex** | 1845 | 0.15 [0.11, 0.19] | < 0.001 |  | 1843 | 0.01 [-0.03, 0.05] | 0.73 |  | 1790 | -0.06 [-0.11, -0.02] | 0.004 |
|  | **FMD% × Sex** | 1845 | 0.01 [-0.03, 0.04] | 0.67 |  | 1843 | -0.03 [-0.06, 0.01] | 0.18 |  | 1790 | 0.01 [-0.02, 0.05] | 0.44 |
| A2: Male | **FMD%** | 843 | -0.01 [-0.03, 0.02] | 0.53 |  | 843 | 0.02 [-0.00, 0.05] | 0.11 |  | 806 | -0.02 [-0.05, 0.01] | 0.15 |
| A3: Female | **FMD%** | 1002 | -0.00 [-0.03, 0.02] | 0.98 |  | 1000 | 0.01 [-0.02, 0.03] | 0.60 |  | 984 | -0.02 [-0.04, 0.01] | 0.19 |
|  |  |  |  |  |  |  |  |  |  |  |  |  |
| B1 | **FMD%** | 1845 | -0.01 [-0.03, 0.01] | 0.16 |  | 1843 | 0.01 [-0.01, 0.03] | 0.43 |  | 1790 | -0.03 [-0.05, -0.00] | 0.023 |
|  | **APOE4** | 1845 | -0.06 [-0.10, -0.02] | 0.001 |  | 1843 | -0.02 [-0.06, 0.02] | 0.35 |  | 1790 | -0.02 [-0.06, 0.03] | 0.44 |
|  | **FMD% × APOE4** | 1845 | 0.05 [0.01, 0.09] | 0.018 |  | 1843 | 0.02 [-0.02, 0.06] | 0.37 |  | 1790 | 0.03 [-0.02, 0.07] | 0.23 |
| B2: APOE4(-) | **FMD%** | 1390 | -0.01 [-0.03, 0.01] | 0.37 |  | 1390 | 0.01 [-0.01, 0.03] | 0.33 |  | 1346 | -0.02 [-0.04, 0.00] | 0.065 |
| B3: APOE4(+) | **FMD%** | 404 | 0.02 [-0.03, 0.06] | 0.45 |  | 402 | 0.02 [-0.02, 0.07] | 0.32 |  | 396 | -0.02 [-0.06, 0.03] | 0.48 |
|  |  |  |  |  |  |  |  |  |  |  |  |  |
| C1 | **FMD%** | 1845 | -0.01 [-0.03, 0.02] | 0.50 |  | 1843 | 0.02 [-0.01, 0.04] | 0.15 |  | 1790 | -0.02 [-0.05, 0.00] | 0.096 |
|  | **CRP** | 1845 | 0.02 [-0.02, 0.05] | 0.33 |  | 1843 | -0.02 [-0.06, 0.02] | 0.24 |  | 1790 | 0.01 [-0.03, 0.05] | 0.57 |
|  | **FMD% × CRP** | 1845 | -0.06 [-0.10, -0.02] | 0.002 |  | 1843 | -0.02 [-0.06, 0.02] | 0.33 |  | 1790 | -0.02 [-0.06, 0.03] | 0.47 |
| C2: CRP<3mg/dl | **FMD%** | 1390 | -0.01 [-0.03, 0.01] | 0.45 |  | 1390 | 0.02 [-0.01, 0.04] | 0.14 |  | 1346 | -0.02 [-0.04, 0.01] | 0.15 |
| C3: CRP≥3mg/dl | **FMD%** | 404 | 0.00 [-0.02, 0.03] | 0.78 |  | 402 | 0.01 [-0.02, 0.04] | 0.61 |  | 396 | -0.01 [-0.04, 0.02] | 0.36 |

Participants with no neuropsychological (NP) testing or only one NP test on or after baseline were excluded. The sample size reflects unique individuals. Generalized linear mixed models (GLMMs) were fitted using the lme4 and lmerTest R packages. Scaled (z-score) flow-mediated dilation (FMD%) was analyzed as a continuous predictor of cognitive performance across three domains (memory, executive function, and language). Interaction effects were examined between FMD% and sex (Model A1), APOE4 status (Model B1), and CRP (Model C1), as well as in stratified analyses by sex (Models A2 and A3), APOE4 status (Models B2 and B3), and CRP (Models C2 and C3). All models were adjusted for baseline age, sex, education, APOE4 status, walk test prior to the FMD exam, SBP, DBP, CVD, hypertension and treatment, diabetes and treatment, smoking, lipid treatment, HDL, triglycerides, fasting glucose, and the time interval between the baseline (Exam 7) assessment of peripheral endothelial function and the last post-baseline NP test.

**Supplementary Table S6. Stratified Analysis of Cognitive function domains and RH**

| Models | Predictor | Memory | | |  | Executive Function | | |  | Language | | |
| --- | --- | --- | --- | --- | --- | --- | --- | --- | --- | --- | --- | --- |
|  |  | **N** | **Beta [95% CI]** | **P value** |  | **N** | **Beta [95% CI]** | **P value** |  | **N** | **Beta [95% CI]** | **P value** |
| A1 | **RH** | 1845 | 0.05 [-0.02, 0.11] | 0.17 |  | 1843 | 0.05 [-0.02, 0.12] | 0.13 |  | 1790 | -0.04 [-0.11, 0.04] | 0.34 |
|  | **Sex** | 1845 | 0.14 [0.10, 0.19] | < 0.001 |  | 1843 | 0.00 [-0.05, 0.05] | 0.91 |  | 1790 | -0.08 [-0.13, -0.03] | 0.002 |
|  | **RH × Sex** | 1845 | -0.02 [-0.06, 0.02] | 0.25 |  | 1843 | -0.02 [-0.06, 0.02] | 0.37 |  | 1790 | 0.03 [-0.02, 0.07] | 0.24 |
| A2: Male | **RH** | 843 | 0.03 [-0.00, 0.06] | 0.093 |  | 843 | 0.03 [-0.01, 0.06] | 0.11 |  | 806 | -0.01 [-0.05, 0.03] | 0.58 |
| A3: Female | **RH** | 1002 | -0.01 [-0.04, 0.02] | 0.67 |  | 1000 | 0.02 [-0.01, 0.05] | 0.24 |  | 984 | 0.01 [-0.02, 0.05] | 0.43 |
|  |  |  |  |  |  |  |  |  |  |  |  |  |
| B1 | **RH** | 1845 | 0.01 [-0.02, 0.03] | 0.66 |  | 1843 | 0.02 [-0.00, 0.05] | 0.074 |  | 1790 | -0.00 [-0.03, 0.03] | 0.89 |
|  | **APOE4** | 1845 | -0.05 [-0.10, -0.01] | 0.017 |  | 1843 | -0.02 [-0.07, 0.03] | 0.38 |  | 1790 | -0.02 [-0.07, 0.03] | 0.38 |
|  | **RH × APOE4** | 1845 | 0.02 [-0.03, 0.07] | 0.38 |  | 1843 | 0.00 [-0.05, 0.05] | 0.97 |  | 1790 | 0.03 [-0.02, 0.08] | 0.21 |
| B2: APOE4(-) | **RH** | 1390 | 0.02 [-0.00, 0.05] | 0.11 |  | 1390 | 0.03 [0.01, 0.06] | 0.02 |  | 1346 | 0.01 [-0.02, 0.04] | 0.51 |
| B3: APOE4(+) | **RH** | 404 | -0.03 [-0.08, 0.02] | 0.22 |  | 402 | -0.01 [-0.06, 0.04] | 0.77 |  | 396 | -0.01 [-0.07, 0.04] | 0.63 |
|  |  |  |  |  |  |  |  |  |  |  |  |  |
| C1 | **FMD%** | 1845 | 0.01 [-0.02, 0.03] | 0.66 |  | 1843 | 0.03 [0.00, 0.06] | 0.028 |  | 1790 | -0.01 [-0.04, 0.02] | 0.68 |
|  | **CRP** | 1845 | 0.00 [-0.04, 0.04] | 0.91 |  | 1843 | -0.02 [-0.07, 0.02] | 0.27 |  | 1790 | 0.01 [-0.04, 0.05] | 0.71 |
|  | **RH × CRP** | 1845 | -0.06 [-0.10, -0.01] | 0.016 |  | 1843 | -0.02 [-0.07, 0.02] | 0.35 |  | 1790 | -0.02 [-0.07, 0.03] | 0.37 |
| C2: CRP<3mg/dl | **RH** | 1390 | -0.00 [-0.03, 0.02] | 0.74 |  | 1390 | 0.03 [-0.00, 0.06] | 0.069 |  | 1346 | -0.01 [-0.05, 0.02] | 0.41 |
| C3: CRP≥3mg/dl | **RH** | 404 | 0.03 [-0.01, 0.06] | 0.13 |  | 402 | 0.02 [-0.02, 0.05] | 0.44 |  | 396 | 0.03 [-0.01, 0.07] | 0.15 |

Participants with no neuropsychological (NP) testing or only one NP test on or after baseline were excluded. The sample size reflects unique individuals. Generalized linear mixed models (GLMMs) were fitted using the lme4 and lmerTest R packages. Scaled (z-score) reactive hyperemia (RH) was analyzed as a continuous predictor of cognitive performance across three domains (memory, executive function, and language). Interaction effects were examined between RH and sex (Model A1), APOE4 status (Model B1), and CRP (Model C1), as well as in stratified analyses by sex (Models A2 and A3), APOE4 status (Models B2 and B3), and CRP (Models C2 and C3). All models were adjusted for baseline age, sex, education, APOE4 status, walk test prior to the RH exam, SBP, DBP, CVD, hypertension and treatment, diabetes and treatment, smoking, lipid treatment, HDL, triglycerides, fasting glucose, and the time interval between the baseline (Exam 7) assessment of peripheral endothelial function and the last post-baseline NP test.

**Supplementary Table S7. Association of plasma biomarkers with the interaction between age and FMD% or RH.**

| Outcomes ^‡^  Blood biomarkers | Predictor ^†^ = FMD% | | | |  | Predictor ^†^ = RH | | | |
| --- | --- | --- | --- | --- | --- | --- | --- | --- | --- |
|  | **Interaction model ^#^** | **N** | **Beta [95% CI]** | **P value** |  | **Interaction model ^#^** | **N** | **Beta [95% CI]** | **P value** |
| Aβ42/aβ40 | **FMD%** | 2720 | 0.01 [-0.03, 0.05] | 0.61 |  | **RH** | 2181 | 0.04 [-0.00, 0.09] | 0.07 |
|  | **Age** | 2720 | -0.11 [-0.15, -0.07] | < 0.001 |  | **Age** | 2181 | -0.09 [-0.14, -0.05] | < 0.001 |
|  | **FMD% × Age** | 2720 | -0.01 [-0.05, 0.03] | 0.62 |  | **RH × Age** | 2181 | -0.00 [-0.05, 0.04] | 0.85 |
| Total Tau | **FMD%** | 1820 | -0.03 [-0.08, 0.02] | 0.25 |  | **RH** | 1432 | -0.08 [-0.14, -0.02] | 0.006 |
|  | **Age** | 1820 | 0.11 [0.05, 0.16] | < 0.001 |  | **Age** | 1432 | 0.10 [0.04, 0.17] | 0.001 |
|  | **FMD% × Age** | 1820 | -0.07 [-0.11, -0.02] | 0.009 |  | **RH × Age** | 1432 | -0.11 [-0.17, -0.05] | < 0.001 |
| pTau_181_ | **FMD%** | 1812 | -0.04 [-0.09, 0.01] | 0.08 |  | **RH** | 1425 | -0.00 [-0.06, 0.05] | 0.92 |
|  | **Age** | 1812 | 0.50 [0.45, 0.55] | < 0.001 |  | **Age** | 1425 | 0.51 [0.45, 0.56] | < 0.001 |
|  | **FMD% × Age** | 1812 | -0.05 [-0.10, -0.01] | 0.028 |  | **RH × Age** | 1425 | -0.03 [-0.08, 0.03] | 0.32 |

† The predictor was one of the measures of brachial artery flow-mediated dilation (FMD%) or reactive hyperemia (RH). ‡ The outcomes were plasma biomarkers (Aβ42/Aβ40, total tau, and pTau₁₈₁), which were log-transformed and rescaled to z-scores. # The interaction term tested between one of predictor (FMD% or RH) age in the multivariate model, where age was scaled to a z-score to harmonize the interaction effect size with all predictors in the interaction terms.

Two-tailed p-values were obtained from multivariate linear regression models—adjusted for sex, education, and APOE4 status for Aβ42/Aβ40 (measured at baseline Exam 7), and adjusted for sex, education, APOE4 status, and the time difference between baseline and the biomarker measurement for total tau and pTau181 (measured at baseline Exam 9).

**Supplementary Table S8. Associations of cerebral microbleeds (CMBs) and white matter hyperintensities (WMHs) with FMD%% and RH in multivariate-adjusted models.**

| Outcomes ^‡^ | Predictor ^†^ = FMD% | | | |  | Predictor ^†^ = RH | | | |
| --- | --- | --- | --- | --- | --- | --- | --- | --- | --- |
|  | **Interaction model ^#^** | **N** | **Beta [95% CI]** | **P value** |  | **Interaction model ^#^** | **N** | **Beta [95% CI]** | **P value** |
| CMB | **FMD%** | 1574 | 0.94 [0.76, 1.17] | 0.60 |  | **RH** | 1213 | 0.91 [0.70, 1.20] | 0.51 |
|  | **Age** | 1574 | 2.08 [1.68, 2.57] | <.001 |  | **Age** | 1213 | 2.03 [1.56, 2.64] | <.001 |
|  | **FMD% × Age** | 1574 | 0.99 [0.80, 1.23] | 0.94 |  | **RH × Age** | 1213 | 0.95 [0.74, 1.23] | 0.69 |
| WMH | **FMD%** | 1503 | -0.03 [-0.08, 0.02] | 0.24 |  | **RH** | 1151 | -0.05 [-0.11, 0.02] | 0.18 |
|  | **Age** | 1503 | 0.35 [0.30, 0.40] | < 0.001 |  | **Age** | 1151 | 0.37 [0.31, 0.44] | < 0.001 |
|  | **FMD% × Age** | 1503 | -0.04 [-0.09, 0.01] | 0.11 |  | **RH × Age** | 1151 | -0.08 [-0.14, -0.02] | 0.008 |

*The outcome variables were CMBs (binary variable, logistic regression model) or WMHs (z-scores of log-transformed continuous variables, linear regression model) in each model.

† The predictor was one of the measures of brachial artery flow-mediated dilation (FMD%%) or reactive hyperemia (RH).

# The interaction term tested was predictor × age in the multivariate model, where age was scaled to a z-score to harmonize the interaction effect size with all predictors in the interaction terms.

Two-tailed p-values were obtained from models adjusted for sex, age, APOE4 status, and the time difference between the MRI scan and the baseline examination.

**Supplementary Table S9. Associations of brain MRI volumes with the interaction between FMD% or RH and age.**

| Outcomes  Brian MRI volumes | Predictor ^†^ = FMD% | | | |  | Predictor ^†^ = RH | | | |
| --- | --- | --- | --- | --- | --- | --- | --- | --- | --- |
|  | **Interaction model** | **N** | **Beta [95% CI]** | **P value** |  | **Interaction model** | **N** | **Beta [95% CI]** | **P value** |
| TCV | **FMD%** | 1992 | -0.03 [-0.06, 0.01] | 0.19 |  | **RH** | 1555 | 0.07 [0.03, 0.12] | 0.001 |
|  | **Age** | 1992 | -0.09 [-0.12, -0.05] | < 0.001 |  | **Age** | 1555 | -0.06 [-0.10, -0.02] | 0.008 |
|  | **FMD% × Age** | 1992 | -0.02 [-0.05, 0.01] | 0.26 |  | **RH × Age** | 1555 | -0.01 [-0.05, 0.03] | 0.65 |
| Lateral vent | **FMD%** | 1992 | 0.01 [-0.03, 0.05] | 0.56 |  | **RH** | 1555 | -0.04 [-0.08, 0.01] | 0.15 |
|  | **Age** | 1992 | 0.48 [0.44, 0.52] | < 0.001 |  | **Age** | 1555 | 0.45 [0.40, 0.50] | < 0.001 |
|  | **FMD% × Age** | 1992 | -0.03 [-0.07, 0.01] | 0.14 |  | **RH × Age** | 1555 | -0.06 [-0.10, -0.02] | 0.006 |
| Third vent | **FMD%** | 1992 | 0.01 [-0.03, 0.05] | 0.56 |  | **RH** | 1555 | -0.01 [-0.06, 0.03] | 0.63 |
|  | **Age** | 1992 | 0.55 [0.51, 0.58] | < 0.001 |  | **Age** | 1555 | 0.54 [0.50, 0.59] | < 0.001 |
|  | **FMD% × Age** | 1992 | -0.02 [-0.05, 0.02] | 0.28 |  | **RH × Age** | 1555 | -0.03 [-0.07, 0.01] | 0.17 |
| Frontal | **FMD%** | 1992 | -0.01 [-0.05, 0.03] | 0.57 |  | **RH** | 1555 | 0.03 [-0.02, 0.07] | 0.24 |
|  | **Age** | 1992 | -0.58 [-0.62, -0.54] | < 0.001 |  | **Age** | 1555 | -0.57 [-0.62, -0.53] | < 0.001 |
|  | **FMD% × Age** | 1992 | 0.04 [0.00, 0.07] | 0.041 |  | **RH × Age** | 1555 | 0.03 [-0.01, 0.07] | 0.17 |
| Occipital | **FMD%** | 1992 | 0.05 [0.00, 0.09] | 0.039 |  | **RH** | 1555 | 0.02 [-0.03, 0.08] | 0.37 |
|  | **Age** | 1992 | -0.27 [-0.31, -0.23] | < 0.001 |  | **Age** | 1555 | -0.26 [-0.31, -0.20] | < 0.001 |
|  | **FMD% × Age** | 1992 | 0.01 [-0.03, 0.05] | 0.73 |  | **RH × Age** | 1555 | -0.02 [-0.06, 0.03] | 0.48 |
| Parietal | **FMD%** | 1992 | -0.03 [-0.07, 0.01] | 0.19 |  | **RH** | 1555 | 0.01 [-0.04, 0.06] | 0.72 |
|  | **Age** | 1992 | -0.37 [-0.41, -0.32] | < 0.001 |  | **Age** | 1555 | -0.35 [-0.40, -0.30] | < 0.001 |
|  | **FMD% × Age** | 1992 | 0.01 [-0.03, 0.06] | 0.49 |  | **RH × Age** | 1555 | 0.06 [0.02, 0.11] | 0.005 |
| Temporal | **FMD%** | 1992 | 0.01 [-0.03, 0.05] | 0.77 |  | **RH** | 1555 | 0.04 [-0.01, 0.09] | 0.12 |
|  | **Age** | 1992 | -0.48 [-0.52, -0.44] | < 0.001 |  | **Age** | 1555 | -0.47 [-0.52, -0.43] | < 0.001 |
|  | **FMD% × Age** | 1992 | 0.02 [-0.02, 0.06] | 0.26 |  | **RH × Age** | 1555 | 0.02 [-0.02, 0.06] | 0.36 |
| Hippocampus | **FMD%** | 1992 | 0.01 [-0.04, 0.05] | 0.81 |  | **RH** | 1555 | -0.00 [-0.06, 0.05] | 0.89 |
|  | **Age** | 1992 | -0.19 [-0.23, -0.14] | < 0.001 |  | **Age** | 1555 | -0.17 [-0.22, -0.12] | < 0.001 |
|  | **FMD% × Age** | 1992 | 0.06 [0.02, 0.10] | 0.003 |  | **RH × Age** | 1555 | 0.05 [0.00, 0.09] | 0.033 |

* The outcome variables were brain MRI volumes (total cranial volume [TCV] was log-transformed and rescaled to z-scores; other measures were also log transformed z-scores after adjusted for head size using TCV) in each model.

† The predictor was one of the measures of brachial artery flow-mediated dilation (FMD%%) or reactive hyperemia (RH).

# The interaction term tested was predictor × age in the multivariate model, where age was scaled to a z-score to harmonize the interaction effect size with all predictors in the interaction terms.

Two-tailed p-values were obtained from models adjusted for sex, age, APOE4 status, and the time difference between the MRI scan and the baseline examination

**Supplementary Table S10. Stratified Analysis of Blood biomarkers and FMD%**

| Models | Predictor | Outcome = Aβ42/aβ40 | | |  | Outcome = Total Tau | | |  | Outcome = pTau_181_ | | |
| --- | --- | --- | --- | --- | --- | --- | --- | --- | --- | --- | --- | --- |
|  |  | **N** | **Beta [95% CI]** | **P value** |  | **N** | **Beta [95% CI]** | **P value** |  | **N** | **Beta [95% CI]** | **P value** |
| A1 | **FMD%** | 2720 | 0.02 [-0.11, 0.16] | 0.73 |  | 1820 | -0.07 [-0.24, 0.09] | 0.37 |  | 1812 | -0.11 [-0.28, 0.06] | 0.19 |
|  | **Sex** | 2720 | 0.05 [-0.03, 0.13] | 0.22 |  | 1820 | 0.12 [0.03, 0.21] | 0.012 |  | 1812 | -0.04 [-0.14, 0.05] | 0.38 |
|  | **FMD% × Sex** | 2720 | -0.01 [-0.09, 0.07] | 0.88 |  | 1820 | 0.06 [-0.03, 0.16] | 0.19 |  | 1812 | 0.08 [-0.02, 0.17] | 0.11 |
| A2: Male | **FMD%** | 1272 | 0.01 [-0.04, 0.07] | 0.65 |  | 832 | -0.02 [-0.07, 0.03] | 0.51 |  | 830 | -0.05 [-0.12, 0.02] | 0.17 |
| A3: Female | **FMD%** | 1448 | 0.02 [-0.04, 0.07] | 0.54 |  | 988 | 0.04 [-0.02, 0.11] | 0.21 |  | 982 | 0.04 [-0.02, 0.11] | 0.20 |
|  |  |  |  |  |  |  |  |  |  |  |  |  |
| B1 | **FMD%** | 2720 | 0.00 [-0.04, 0.05] | 0.95 |  | 1820 | -0.01 [-0.06, 0.05] | 0.77 |  | 1812 | 0.02 [-0.03, 0.08] | 0.38 |
|  | **APOE4** | 2720 | -0.15 [-0.24, -0.06] | 0.001 |  | 1820 | 0.07 [-0.05, 0.18] | 0.24 |  | 1812 | -0.01 [-0.13, 0.10] | 0.81 |
|  | **FMD% × APOE4** | 2720 | 0.06 [-0.03, 0.15] | 0.28 |  | 1820 | 0.17 [0.06, 0.28] | 0.002 |  | 1812 | -0.02 [-0.13, 0.08] | 0.66 |
| B2: APOE4(-) | **FMD%** | 2118 | 0.00 [-0.04, 0.05] | 0.95 |  | 1428 | -0.01 [-0.06, 0.05] | 0.79 |  | 1422 | 0.02 [-0.03, 0.08] | 0.41 |
| B3: APOE4(+) | **FMD%** | 602 | 0.07 [-0.02, 0.15] | 0.12 |  | 392 | 0.07 [-0.03, 0.17] | 0.19 |  | 390 | -0.02 [-0.12, 0.07] | 0.61 |
|  |  |  |  |  |  |  |  |  |  |  |  |  |
| C1 | **FMD%** | 2720 | 0.04 [-0.02, 0.09] | 0.18 |  | 1820 | 0.05 [-0.01, 0.11] | 0.08 |  | 1812 | 0.05 [-0.01, 0.11] | 0.12 |
|  | **CRP** | 2720 | -0.10 [-0.17, -0.02] | 0.014 |  | 1820 | 0.01 [-0.09, 0.11] | 0.87 |  | 1812 | -0.01 [-0.11, 0.09] | 0.79 |
|  | **FMD% × CRP** | 2720 | -0.05 [-0.13, 0.02] | 0.18 |  | 1820 | -0.06 [-0.15, 0.03] | 0.21 |  | 1812 | -0.08 [-0.17, 0.02] | 0.11 |
| C2: CRP<3mg/dl | **FMD%** | 1605 | 0.02 [-0.03, 0.08] | 0.35 |  | 1146 | 0.04 [-0.02, 0.10] | 0.19 |  | 1141 | 0.04 [-0.02, 0.10] | 0.19 |
| C3: CRP≥3mg/dl | **FMD%** | 1115 | -0.01 [-0.07, 0.05] | 0.81 |  | 674 | 0.00 [-0.07, 0.08] | 0.96 |  | 671 | -0.04 [-0.12, 0.03] | 0.29 |

The outcomes variables are aβ42/aβ40, Total Tau and pTau181, respectively. Multivariable linear models were used to estimate β coefficients and 95% confidence intervals (95% CIs) for the interaction effects between FMD% and sex (Model A1), APOE4 status (Model B1), and CRP (Model C1), as well as for stratified analyses by sex (Models A2 and A3), APOE4 status (Models B2 and B3), and CRP (Models C2 and C3). For all interaction models, both the main effects and interaction effect were reported for the two interaction variables. Both FMD% and the outcome variables (Aβ42/Aβ40, total tau, and p-tau181) were standardized as z-scores. All models were adjusted for baseline age, sex, education, and APOE4 status.

**Supplementary Table S11. Stratified Analysis of Blood biomarkers and RH**

| Models | Predictor | Outcome = Aβ42/aβ40 | | |  | Outcome = Total Tau | | |  | Outcome = pTau_181_ | | |
| --- | --- | --- | --- | --- | --- | --- | --- | --- | --- | --- | --- | --- |
|  |  | **N** | **Beta [95% CI]** | **P value** |  | **N** | **Beta [95% CI]** | **P value** |  | **N** | **Beta [95% CI]** | **P value** |
| A1 | **RH** | 2181 | 0.01 [-0.13, 0.15] | 0.87 |  | 1432 | -0.12 [-0.32, 0.08] | 0.24 |  | 1425 | -0.04 [-0.24, 0.17] | 0.73 |
|  | **Sex** | 2181 | 0.06 [-0.02, 0.14] | 0.16 |  | 1432 | 0.09 [-0.03, 0.21] | 0.13 |  | 1425 | -0.04 [-0.16, 0.08] | 0.53 |
|  | **RH × Sex** | 2181 | 0.02 [-0.07, 0.10] | 0.67 |  | 1432 | 0.11 [-0.01, 0.23] | 0.07 |  | 1425 | 0.04 [-0.08, 0.17] | 0.47 |
| A2: Male | **RH** | 996 | 0.01 [-0.05, 0.08] | 0.71 |  | 643 | -0.02 [-0.09, 0.04] | 0.49 |  | 642 | 0.01 [-0.08, 0.10] | 0.88 |
| A3: Female | **RH** | 1185 | 0.06 [-0.01, 0.12] | 0.07 |  | 789 | 0.08 [-0.01, 0.16] | 0.09 |  | 783 | 0.05 [-0.04, 0.14] | 0.31 |
|  |  |  |  |  |  |  |  |  |  |  |  |  |
| B1 | **RH** | 2181 | 0.03 [-0.02, 0.08] | 0.29 |  | 1432 | -0.01 [-0.08, 0.06] | 0.71 |  | 1425 | 0.04 [-0.04, 0.11] | 0.32 |
|  | **APOE4** | 2181 | -0.17 [-0.27, -0.07] | < 0.001 |  | 1432 | 0.08 [-0.06, 0.22] | 0.24 |  | 1425 | -0.02 [-0.16, 0.12] | 0.77 |
|  | **RH × APOE4** | 2181 | 0.06 [-0.04, 0.16] | 0.24 |  | 1432 | 0.33 [0.19, 0.47] | < 0.001 |  | 1425 | -0.01 [-0.16, 0.14] | 0.91 |
| B2: APOE4(-) | **RH** | 1691 | 0.03 [-0.02, 0.08] | 0.27 |  | 1116 | -0.03 [-0.09, 0.03] | 0.36 |  | 1111 | 0.03 [-0.04, 0.11] | 0.36 |
| B3: APOE4(+) | **RH** | 490 | 0.08 [-0.01, 0.17] | 0.09 |  | 316 | 0.14 [0.00, 0.28] | 0.048 |  | 314 | 0.10 [-0.01, 0.21] | 0.06 |
|  |  |  |  |  |  |  |  |  |  |  |  |  |
| C1 | **RH** | 2181 | 0.04 [-0.02, 0.10] | 0.21 |  | 1432 | 0.10 [0.02, 0.18] | 0.016 |  | 1425 | 0.05 [-0.03, 0.14] | 0.20 |
|  | **CRP** | 2181 | -0.09 [-0.17, -0.00] | 0.047 |  | 1432 | 0.01 [-0.11, 0.14] | 0.83 |  | 1425 | -0.02 [-0.15, 0.10] | 0.75 |
|  | **RH × CRP** | 2181 | -0.00 [-0.08, 0.08] | 0.99 |  | 1432 | -0.11 [-0.23, 0.01] | 0.07 |  | 1425 | -0.05 [-0.17, 0.07] | 0.44 |
| C2: CRP<3mg/dl | **RH** | 1280 | 0.02 [-0.04, 0.08] | 0.48 |  | 902 | 0.08 [-0.00, 0.16] | 0.06 |  | 898 | 0.05 [-0.04, 0.13] | 0.28 |
| C3: CRP≥3mg/dl | **RH** | 901 | 0.06 [-0.01, 0.14] | 0.09 |  | 530 | -0.02 [-0.12, 0.07] | 0.62 |  | 527 | 0.02 [-0.08, 0.11] | 0.75 |

The outcomes variables are aβ42/aβ40, Total Tau and pTau181, respectively. Multivariable linear models were used to estimate β coefficients and 95% confidence intervals (95% CIs) for the interaction effects between RH and sex (Model A1), APOE4 status (Model B1), and CRP (Model C1), as well as for stratified analyses by sex (Models A2 and A3), APOE4 status (Models B2 and B3), and CRP (Models C2 and C3). For all interaction models, both the main effects and interaction effect were reported for the two interaction variables. Both RH and the outcome variables (Aβ42/Aβ40, total tau, and p-tau181) were standardized as z-scores. All models were adjusted for baseline age, sex, education, and APOE4 status.

**Supplementary Table S12. Stratified Analysis of Brain MRI and FMD%**

| Models | Predictor | Outcome = CMB | | |  | Outcome = WMH | | |  | Outcome = Hippocampus | | |
| --- | --- | --- | --- | --- | --- | --- | --- | --- | --- | --- | --- | --- |
|  |  | **N** | **OR [95% CI]** | **P value** |  | **N** | **Beta [95% CI]** | **P value** |  | **N** | **Beta [95% CI]** | **P value** |
| A1 | **FMD%** | 1574 | 1.15 [0.59, 2.22] | 0.69 |  | 1496 | 0.04 [-0.13, 0.21] | 0.64 |  | 1992 | 0.00 [-0.14, 0.15] | 0.99 |
|  | **Sex** | 1574 | 0.59 [0.40, 0.85] | 0.005 |  | 1496 | 0.09 [-0.01, 0.18] | 0.08 |  | 1992 | 0.32 [0.24, 0.40] | < 0.001 |
|  | **FMD% × Sex** | 1574 | 0.88 [0.58, 1.33] | 0.55 |  | 1496 | 0.03 [0.01, 0.05] | 0.016 |  | 1992 | -0.01 [-0.02, 0.01] | 0.51 |
| A2: Male | **FMD%** | 739 | 1.01 [0.77, 1.31] | 0.96 |  | 705 | 0.00 [-0.06, 0.07] | 0.98 |  | 950 | -0.01 [-0.07, 0.05] | 0.72 |
| A3: Female | **FMD%** | 835 | 0.90 [0.65, 1.24] | 0.52 |  | 791 | -0.04 [-0.12, 0.03] | 0.27 |  | 1042 | -0.01 [-0.07, 0.05] | 0.71 |
|  |  |  |  |  |  |  |  |  |  |  |  |  |
| B1 | **FMD%** | 1574 | 0.91 [0.71, 1.16] | 0.46 |  | 1496 | -0.03 [-0.09, 0.03] | 0.31 |  | 1992 | -0.02 [-0.07, 0.02] | 0.33 |
|  | **APOE4** | 1574 | 1.03 [0.66, 1.60] | 0.89 |  | 1496 | -0.01 [-0.12, 0.11] | 0.9 |  | 1992 | -0.06 [-0.15, 0.04] | 0.26 |
|  | **FMD% × APOE4** | 1574 | 1.15 [0.72, 1.83] | 0.57 |  | 1496 | 0.02 [-0.09, 0.13] | 0.72 |  | 1992 | 0.05 [-0.05, 0.14] | 0.35 |
| B2: APOE4(-) | **FMD%** | 1205 | 0.90 [0.70, 1.14] | 0.38 |  | 1154 | -0.03 [-0.09, 0.03] | 0.31 |  | 1545 | -0.02 [-0.07, 0.02] | 0.34 |
| B3: APOE4(+) | **FMD%** | 369 | 1.11 [0.71, 1.73] | 0.66 |  | 342 | -0.00 [-0.09, 0.08] | 0.95 |  | 447 | 0.01 [-0.08, 0.11] | 0.80 |
|  |  |  |  |  |  |  |  |  |  |  |  |  |
| C1 | **FMD%** | 1574 | 0.85 [0.64, 1.13] | 0.26 |  | 1496 | -0.02 [-0.08, 0.05] | 0.64 |  | 1992 | -0.02 [-0.07, 0.04] | 0.51 |
|  | **CRP** | 1574 | 1.20 [0.82, 1.76] | 0.34 |  | 1496 | -0.08 [-0.18, 0.02] | 0.13 |  | 1992 | -0.01 [-0.09, 0.08] | 0.84 |
|  | **FMD% × CRP** | 1574 | 1.26 [0.84, 1.91] | 0.27 |  | 1496 | -0.02 [-0.12, 0.08] | 0.65 |  | 1992 | 0.01 [-0.07, 0.10] | 0.76 |
| C2: CRP<3mg/dl | **FMD%** | 992 | 0.85 [0.64, 1.13] | 0.27 |  | 957 | -0.01 [-0.07, 0.06] | 0.86 |  | 1219 | -0.02 [-0.08, 0.04] | 0.48 |
| C3: CRP≥3mg/dl | **FMD%** | 582 | 1.05 [0.76, 1.45] | 0.76 |  | 539 | -0.05 [-0.13, 0.02] | 0.15 |  | 773 | -0.00 [-0.07, 0.06] | 0.89 |

The outcome variables were CMB, WMH, and hippocampal volume (brain MRI–derived). Multivariable logistic regression models were used to estimate odds ratios (ORs) and 95% confidence intervals (95% CIs) for the binary outcome CMB, and multivariable linear regression models were used for the continuous outcomes (WMH and hippocampal volume) to estimate β coefficients and 95% CIs for the interaction effects between FMD% and sex (Model A1), APOE4 status (Model B1), and CRP (Model C1), as well as for stratified analyses by sex (Models A2 and A3), APOE4 status (Models B2 and B3), and CRP (Models C2 and C3). For all interaction models, both the main effects and the interaction effect were reported for the two interacting variables. WMH and hippocampal volumes were adjusted for head size by dividing by total cranial volume. The continuous variables FMD%, WMH, and hippocampal volume were standardized as z-scores. All models were adjusted for baseline age, sex, education, APOE4 status, and the time difference between baseline and MRI dates.

**Supplementary Table S13. Stratified Analysis of Brain MRI and RH**

| Models | Predictor | Outcome = CMB | | |  | Outcome = WMH | | |  | Outcome = Hippocampus | | |
| --- | --- | --- | --- | --- | --- | --- | --- | --- | --- | --- | --- | --- |
|  |  | **N** | **OR [95% CI]** | **P value** |  | **N** | **Beta [95% CI]** | **P value** |  | **N** | **Beta [95% CI]** | **P value** |
| A1 | **RH** | 1213 | 1.17 [0.56, 2.45] | 0.67 |  | 1145 | -0.04 [-0.24, 0.16] | 0.68 |  | 1555 | 0.03 [-0.12, 0.19] | 0.69 |
|  | **Sex** | 1213 | 0.56 [0.36, 0.87] | 0.01 |  | 1145 | 0.08 [-0.03, 0.20] | 0.16 |  | 1555 | 0.35 [0.26, 0.45] | < 0.001 |
|  | **RH × Sex** | 1213 | 0.84 [0.52, 1.35] | 0.46 |  | 1145 | 0.03 [0.00, 0.06] | 0.022 |  | 1555 | -0.00 [-0.02, 0.01] | 0.69 |
| A2: Male | **RH** | 556 | 0.95 [0.68, 1.33] | 0.77 |  | 525 | -0.05 [-0.14, 0.04] | 0.28 |  | 724 | 0.01 [-0.07, 0.08] | 0.87 |
| A3: Female | **RH** | 657 | 0.83 [0.55, 1.26] | 0.38 |  | 620 | -0.02 [-0.12, 0.08] | 0.67 |  | 831 | -0.02 [-0.09, 0.05] | 0.61 |
|  |  |  |  |  |  |  |  |  |  |  |  |  |
| B1 | **RH** | 1213 | 0.95 [0.71, 1.26] | 0.72 |  | 1145 | -0.04 [-0.12, 0.03] | 0.26 |  | 1555 | -0.02 [-0.07, 0.04] | 0.55 |
|  | **APOE4** | 1213 | 0.90 [0.52, 1.55] | 0.70 |  | 1145 | -0.01 [-0.15, 0.12] | 0.84 |  | 1555 | -0.06 [-0.17, 0.05] | 0.29 |
|  | **RH × APOE4** | 1213 | 0.78 [0.42, 1.43] | 0.42 |  | 1145 | 0.04 [-0.10, 0.19] | 0.57 |  | 1555 | 0.02 [-0.09, 0.13] | 0.69 |
| B2: APOE4(-) | **RH** | 918 | 0.94 [0.70, 1.26] | 0.68 |  | 874 | -0.05 [-0.13, 0.03] | 0.24 |  | 1202 | -0.01 [-0.07, 0.04] | 0.66 |
| B3: APOE4(+) | **RH** | 295 | 0.80 [0.44, 1.47] | 0.47 |  | 271 | 0.01 [-0.11, 0.12] | 0.89 |  | 353 | -0.02 [-0.14, 0.10] | 0.77 |
|  |  |  |  |  |  |  |  |  |  |  |  |  |
| C1 | **RH** | 1213 | 0.82 [0.59, 1.14] | 0.24 |  | 1145 | -0.06 [-0.15, 0.02] | 0.13 |  | 1555 | -0.01 [-0.08, 0.05] | 0.65 |
|  | **CRP** | 1213 | 1.20 [0.76, 1.88] | 0.43 |  | 1145 | -0.10 [-0.23, 0.02] | 0.10 |  | 1555 | -0.05 [-0.15, 0.04] | 0.28 |
|  | **RH × CRP** | 1213 | 1.28 [0.80, 2.07] | 0.31 |  | 1145 | 0.07 [-0.05, 0.19] | 0.26 |  | 1555 | 0.00 [-0.09, 0.09] | 0.98 |
| C2: CRP<3mg/dl | **RH** | 764 | 0.85 [0.61, 1.20] | 0.37 |  | 730 | -0.05 [-0.14, 0.04] | 0.26 |  | 945 | -0.01 [-0.08, 0.06] | 0.74 |
| C3: CRP≥3mg/dl | **RH** | 449 | 1.01 [0.67, 1.53] | 0.96 |  | 415 | -0.01 [-0.11, 0.08] | 0.81 |  | 610 | -0.02 [-0.11, 0.06] | 0.55 |

The outcome variables were CMB, WMH, and hippocampal volume (brain MRI–derived). Multivariable logistic regression models were used to estimate odds ratios (ORs) and 95% confidence intervals (95% CIs) for the binary outcome CMB, and multivariable linear regression models were used for the continuous outcomes (WMH and hippocampal volume) to estimate β coefficients and 95% CIs for the interaction effects between FMD% and sex (Model A1), APOE4 status (Model B1), and CRP (Model C1), as well as for stratified analyses by sex (Models A2 and A3), APOE4 status (Models B2 and B3), and CRP (Models C2 and C3). For all interaction models, both the main effects and the interaction effect were reported for the two interacting variables. WMH and hippocampal volumes were adjusted for head size by dividing by total cranial volume. The continuous variables FMD%, WMH, and hippocampal volume were standardized as z-scores. All models were adjusted for baseline age, sex, education, APOE4 status, and the time difference between baseline and MRI dates.
